# Intramolecular Synergy of CO_2_ Activation and H Spillover on Heteronuclear Dual‐Metal Phthalocyanine Assemblies for Selective CO_2_ Photoreduction

**DOI:** 10.1002/advs.202521954

**Published:** 2026-01-04

**Authors:** Ye Liu, Wei Qin, Panzhe Qiao, Ziqing Zhang, Zhuo Li, Yangyang Zhu, Jianhui Sun, Zhijun Li, Fuquan Bai, Liqiang Jing, Ji Bian

**Affiliations:** ^1^ Key Laboratory of Functional Inorganic Materials Chemistry (Ministry of Education) School of Chemistry and Materia ls Science International Joint Research Center and Lab for Catalytic Technology Heilongjiang University Harbin 150080 P. R. China; ^2^ International Joint Research Laboratory of Nano‐Micro Architecture Chemistry Institute of Theoretical Chemistry Jilin University Changchun 130021 P. R. China; ^3^ Shanghai Synchrotron Radiation Facility Shanghai Advanced Research Institute Chinese Academy of Sciences Shanghai 201210 P. R. China

**Keywords:** dual‐atom sites, heteronuclear metal phthalocyanine assemblies, photocatalysis, ultrafast electron kinetics, CO_2_ conversion, Z‐scheme charge transfer

## Abstract

Solar‐driven CO_2_ conversion holds great promise in carbon recycling. CO_2_ activation and hydrogen spillover are crucial for high‐selectivity CO_2_ reduction, while with great challenges. Here, heteronuclear metal phthalocyanine aggregates with atomically active sites are synthesized and then assembled on BiVO_4_ nanosheets. The CuNiPc/BiVO_4_ nanocomposite achieves a 238 mmol g_Cu_^−1^ h^−1^ CO yield with nearly 100% selectivity (vs 77% for mononuclear CuPc/BiVO_4_) without H_2_ evolution, ranking among top atomic‐engineered photocatalysts. Femtosecond‐transient absorption spectra, in situ synchrotron radiation measurements, and theoretical simulations, etc., reveal that such a difference is mainly ascribed to the fast interfacial Z‐scheme charge transfer kinetics and the synergy catalysis between dual sites in CuNiPc. The Cu–N_4_ moiety enhances CO_2_ adsorption and activation relative to CuPc due to the regulated Cu configuration caused by the Ni atom incorporation, while the adjacent Ni–N_4_ unit activates H_2_O to generate ^*^H, which subsequently undergoes intramolecular spillover to ^*^Cu–COO site, consequently accessing both CO_2_ activation and protonation for ^*^COOH generation towards highly selective CO_2_ reduction.

## Graphical abstract

The CuNiPc/BiVO_4_ heterojunction is designed and synthesized for selective CO_2_ photoreduction. The cascade interfacial Z‐scheme charge transfer and the intramolecular synergy of Cu–Ni dual sites on CO_2_ activation and H spillover contribute to the high photocatalytic performance and selectivity.

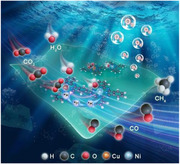

## 1 Introduction

Solar‐driven CO_2_ reduction into chemical fuels is an ideal strategy to access carbon neutrality [1]. For the past few years, substantial efforts have been devoted to seeking promising photocatalysts and engineering catalytically active sites for CO_2_ reduction [2]. In recent decades, metal phthalocyanines (MPcs) and derivatives have been an emerging material in CO_2_ conversion with the merits of the metal–N_4_ (M–N_4_) moiety as the isolated active site [3]. The extraction of the electrons in MPcs as much as possible is the key to initiating such a reaction. Our pioneering study [4] and the following work [5, 6, 7] established that employing MPcs as a reduction composite and coupling with an oxidative semiconductor to construct a Z‐scheme heterojunction is a feasible strategy. This is because the photogenerated electrons of MPcs can access spatial separation with the photogenerated holes on the oxidative counterpart, and the high redox potentials can also be preserved simultaneously to drive the overall CO_2_ reduction reactions. However, the obtained photocatalytic activity is still moderate as the intrinsic electronic configurations of the M–N_4_ site would hardly break the stubborn restriction of CO_2_ activation and catalytic performance [8].

For initiating CO_2_ reduction reactions, two distinct pathways are commonly considered. One pathway is the proton‐coupled electron transfer (PCET) process, in which CO_2_ adsorption/activation typically occurs at a single metal site [9]. Alternatively, a thermodynamically favorable hydrogen‐mediated pathway for CO_2_ reduction had been proposed [10]. In this case, H_2_O activation/dissociation on the catalyst surface generates active hydrogen species (^*^H) and then initiates CO_2_ activation, yet it is challenging to achieve such a reaction at a single active site due to the complex coordination. It is noteworthy that two pathways critically depend on efficient CO_2_ activation and proton supply, which are strongly influenced by the chemical environment of active sites and the hydrogen spillover. As widely recognized, hydrogen spillover enables the dynamic migration of ^*^H species across different active sites for enhancing catalytic synergy [11]. Therefore, the rational design of catalysts with spatially separated dual‐active sites at an optimal distance is highly desirable. Such architectures can concurrently promote CO_2_ activation, facilitate H_2_O dissociation to generate ^*^H, and enable efficient hydrogen spillover, possibly significantly improving catalytic performance. Fortunately, the structure of MPcs molecular is highly tailorable at the molecular level [12]. Thus, it is speculated that the properties of MPcs can be further regulated by manipulating the skeleton structure, electronic states, and the coordination environment of the M–N_4_ moiety.

Binuclear metal phthalocyanines (bi‐MPcs), a specific class of phthalocyanine derivatives that are formed when two phthalocyanine monomers share a common benzene ring [13], is standing out as a new brand of catalyst that fulfills the assumption regarding to the above‐mentioned microstructure design and regulation. On the one hand, in comparison to mononuclear metal phthalocyanines (mono‐MPcs), the extension of the π‐conjugated structure brings new opportunities in photocatalysis. The band gap between the highest occupied molecular orbital (HOMO) and the lowest unoccupied molecular orbital (LUMO) decreases in bi‐MPcs due to the conjugated macrocyclic structural features [14]. It means that the electrons can be more easily excited to higher energy levels, thus being more favorable to initiate the reduction reactions [15]. In parallel, the electrical conductivity and charge transfer capabilities can also be improved due to the extensive π‐delocalization and extended conjugated system in bi‐MPcs molecules [16]. Additionally, such π‐extended MPcs also brings the enhanced light harvesting due to the conjugation effects of chromophores [17]. On the other hand, the delocalized π electrons of the phthalocyanine ligands can greatly interact with the d‐orbitals of the central metal atom, consequently influencing the electronic structure of the central metal [18]. Such a different electronic structure of the central metal in bi‐MPcs would present preferable catalytic properties in contrast to mono‐MPcs, benefiting from the adjustable electron density of the M–N_4_ moiety [19]. Significantly, the spin state of metal in the M_1_–N_4_ site can be regulated by introducing the other M_2_–N_4_ site, and the long‐range interactions of the dual sites would lead to the optimization of reactants adsorption/activation [3]. In addition, the catalytic properties of bi‐MPcs can be delicately tuned by varying the metal atoms that are embedded into the cavity structure of aromatic macrocycles. In this case, rational design of bi‐MPc featuring CO_2_ activation, H_2_O activation, and the accompanying H spillover is expected to access highly selective CO_2_ reduction.

To date, Cu remains the most promising active center for CO_2_ adsorption and activation due to the strong hybridization of Cu 3d and CO_2_‐O 2p orbitals, which is quite favorable for promoting the electron transfer from Cu to CO_2_ [20], thus optimizing the rate‐limiting step for CO_2_ reduction. As for the other aspect, Ni species show great potential in activating water molecules to produce protons and accelerating the protonation of adsorbed CO_2_ during the CO_2_ reduction reaction (CO_2_RR) [10]. Thus, the tailored bi‐MPc architecture, featuring well‐defined Cu–N_4_ and Ni–N_4_ moieties, is anticipated to improve photocatalytic performance and selectivity. Crucially, molecular‐scale H spillover occurs more readily on the dual‐site catalyst with appropriate atomic distance than on its mononuclear counterpart [21], thereby providing another thermodynamically favorable route for CO_2_ activation along with proton relay to generate key intermediates of ^*^COOH. Nonetheless, designing and employing such bi‐MPcs as the potential reductive constitute toward CO_2_RR still keep a research blank with great challenges. For the oxidative counterpart, BiVO_4_’s deep valence band position [22] makes it ideally suited for Z‐scheme heterojunctions, as it thermodynamically favors water oxidation half‐reaction in the overall photocatalytic CO_2_RR. The integration of planar bi‐MPcs with 2D BiVO_4_ nanosheets is speculated to form an intimate interface, facilitating charge transfer and then enabling efficient photocatalysis. Crucially, elucidating the interfacial charge transfer manner and kinetics in such a novel system as well as exploring the underlying mechanism on the intrinsic excitation states of bi‐MPcs, is beneficial to unlock the potential advantages of bimetallic architectures over the mono‐MPcs counterpart in CO_2_RR. Femtosecond transient absorption spectroscopy (fs‐TAS) has been a powerful tool to give information on the photophysical processes occurring at ultrafast timescales for organic semiconductors [23]. Specifically, it is feasible to distinguish and explore the kinetics of charge relaxation, transfer, and the injection process on the established Z‐scheme heterojunction with CuNiPc as the reduction constituent. Unfortunately, the in‐depth understanding on the ultrafast photophysical procedure involved in the proposed system still remains a blank in the current research from both the perspective of experiments and theoretical simulation.

Herein, we design and synthesize a heteronuclear metal phthalocyanine CuNiPc and then assemble them on the oxidative semiconductor BiVO_4_ nanosheet to construct Z‐scheme heterojunctions, achieving 100% selectivity towards CO_2_ reduction without H_2_ evolution. The optimal CuNiPc/BiVO_4_ nanocomposite delivers a CO yield rate of 238 mmol g_Cu_^−1^ h^−1^ with nearly 100% selectivity (vs 77% for mononuclear CuPc/BiVO_4_) without H_2_ generation. The introduction of heteronuclear CuNiPc not only accelerates the interfacial Z‐scheme charge transfer kinetics but also displays the synergistic catalytic effect of atomically dual active sites. The Cu–N_4_ unit facilitates the adsorption and activation of CO_2_, while the Ni–N_4_ unit promotes H_2_O dissociation to release active ^*^H. Subsequently, the generated H^*^ undergoes intramolecular spillover to nearby ^*^Cu–COO site, accessing both CO_2_ activation and protonation for ^*^COOH generation. Additionally, the ^*^CO binding on Cu–N_4_ unit is weakened, facilitating CO desorption and enhancing CO selectivity. This work provides an avenue to tailor the catalysis on metal phthalocyanine from molecular level for artificial photosynthesis.

## 2 Results

### 2.1 Synthesis and Structural Characterization

A hydroxyl‐induced assembly strategy is adopted to fabricate CuNiPc modified BiVO_4_ nanosheet (CuNiPc/BiVO_4_), during which CuNiPc is pre‐prepared through a conventional DBU (1,8‐diazabicyclo [5.4.0] undec‐7‐ene) liquid‐phase catalytic method [24] with phthalonitrile, CuCl_2_, and NiCl_2_ as the building blocks, and 1,2,4,5‐tetracyanobenzene as the linker, respectively (Figure S1), while BiVO_4_ nanosheets are synthesized following a previously reported protocol [25]. Other binuclear phthalocyanines modified BiVO_4_ nanosheets were synthesized using the same method, except for the replacement of the corresponding metal salts. In parallel, the mononuclear phthalocyanines have also been synthesized as a contrast (Figure S2). The characterizations and structure information of the obtained binuclear (CuNiPc and CuCuPc) and mononuclear (CuPc) phthalocyanines were validated by Raman spectra, Fourier‐transform infrared (FT‐IR) spectra, and UV–vis absorption spectra, etc (Figures S3–S7).

**FIGURE 1:**
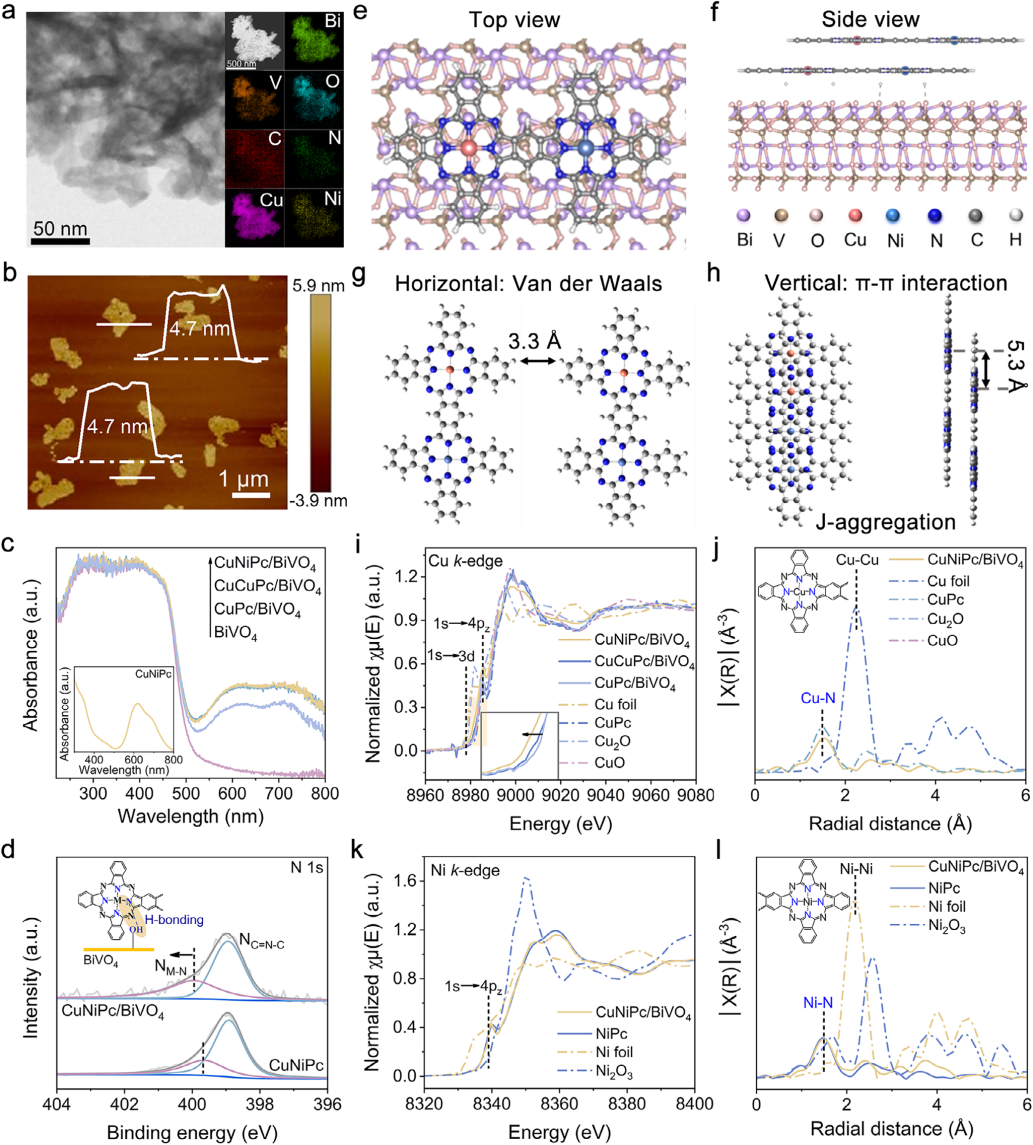
(a) TEM image of CuNiPc/BiVO_4_ and the corresponding EDX mapping images of elemental C, N, Cu, Ni, Bi, V, and O. (b) AFM image and the corresponding height profiles of CuNiPc/BiVO_4_. (c) UV–vis absorption spectra of CuNiPc/BiVO_4_, CuCuPc/BiVO_4_, CuPc/BiVO_4_, and BiVO_4_ within CuNiPc as an inset. (d) XPS analyses for N 1s of CuNiPc and CuNiPc/BiVO_4_. (e) Top and (f) side view of the DFT optimized CuNiPc/BiVO_4_ heterojunction model. The molecular stacking configurations of CuNiPc exhibit both (g) horizontal and (h) vertical orientations. (i) Normalized Cu K‐edge XANES spectra of CuNiPc/BiVO_4_, CuCuPc/BiVO_4_, CuPc/BiVO_4_, and (j) corresponding FT‐EXAFS spectra of CuNiPc/BiVO_4_. (k) Normalized Ni K‐edge XANES spectra and (l) corresponding FT‐EXAFS spectra of CuNiPc/BiVO_4_.

The morphologies of pristine BiVO_4_ and CuNiPc‐modified one were investigated by transmission electron microscopy (TEM) (Figure 1a; Figure S8). One can see the amorphous CuNiPc is uniformly dispersed on the BiVO_4_ nanosheets, and an interplanar spacing of 0.26 nm, corresponding to the (200) planes of BiVO_4_ [26] is observed (Figure S9). The uniform distribution of Bi, V, O, Cu, Ni, C, and N elements on the entire CuNiPc/BiVO_4_ heterojunction has also been detected by the energy dispersive X‐ray spectroscopy (EDX) mapping, demonstrating the successful integration of CuNiPc and BiVO_4_.

As revealed by AFM images and the corresponding height profiles, the thickness of the loaded CuNiPc on BiVO_4_ is determined to be 0.8 nm (Figure 1b; Figure S10). Given that the interlayer distance is 0.38 nm in phthalocyanines [27], it can be inferred that approximately 2 layers of CuNiPc cover the BiVO_4_ nanosheets. The as‐fabricated BiVO_4_ matches well with the monoclinic phase (JCPDS No. 14–0688) [28]. No diffraction peaks attributed to phthalocyanines are observed over the CuNiPc/BiVO_4_, CuCuPc/BiVO_4_, and CuPc/BiVO_4_ (Figure S11), which might be related to the tiny loadings and the ultrathin structure of the assembled phthalocyanines. However, the typical optical absorption of phthalocyanine was detected by UV–vis diffuse reflectance spectroscopy (DRS). The heterojunctions exhibit distinct Q band absorption features between 600 and 800 nm, corresponding to the HOMO‐LUMO transitions of phthalocyanines (Figure 1c) [29]. Obviously, the visible light‐harvesting capability of heterojunctions is extended as the modification of phthalocyanines. Notably, CuNiPc/BiVO_4_ and CuCuPc/BiVO_4_ exhibit enhanced light absorption compared to CuPc/BiVO_4_, which can be attributed to extended π‐conjugation in bi‐MPcs [30].

The interaction between BiVO_4_ and CuNiPc was then explored by Raman spectra (Figure S12). Raman bands at 325, 365, and 827 cm^−1^ are assigned to the asymmetric and symmetric deformation modes of the VO_4_^3−^ tetrahedron as well as the stretching mode of V─O bonds in BiVO_4_, respectively [22]. Notably, the peak assigned to V─O stretching vibration of BiVO_4_ exhibits a slight redshift after the modification with CuNiPc, indicating the successful integration of CuNiPc and BiVO_4_. Moreover, FT‐IR spectra were conducted to uncover the interfacial interactions between CuNiPc and BiVO_4_. As shown in Figure S13, all the characteristic peaks assigned to CuNiPc are detected in CuNiPc/BiVO_4_, indicating the CuNiPc is introduced in the heterojunction. It's worth noting that the peak intensity corresponding to surface hydroxyl groups (1645 cm^−1^) [31] over CuNiPc/BiVO_4_ decreases distinctly in comparison to BiVO_4_, implying that CuNiPc interacts with BiVO_4_ via the surface hydroxyl. Furthermore, the binding energies (BE) of both Bi 4f and V 2p in CuNiPc/BiVO_4_ present a negative shift in comparison to those of bare BiVO_4_ (Figure S14), resulting from the electron diffusion from CuNiPc to BiVO_4_ as they come into close attachment. The N 1s XPS profile of CuNiPc/BiVO_4_ displays two characteristic peaks corresponding to the meta–N and C─N═C, respectively (Figure 1d) [32]. Notably, the metal–N peak exhibits a positive BE shift relative to pristine CuNiPc, whereas the BEs of Cu 2p and Ni 2p remain unshifted (Figure S15). These observations suggest that CuNiPc plausibly links with BiVO_4_ through hydrogen bonding interactions involving the ligand N atoms. The inference is further supported by density functional theory (DFT) calculations (Figure 1e,f), which reveal stable binding between CuNiPc and BiVO_4_ via N─H···O hydrogen bonds with characteristic bond lengths of 1.58 Å (N─H) and 2.1 Å (O─H). Additional simulations examined the stacking behavior of CuNiPc assemblies and their intermolecular interactions in ethanol. In the horizontal orientation, phthalocyanine molecules interact primarily via van der Waals forces, with a fixed spacing of 3.3 Å between monomer units (Figure 1g). While the vertical alignment facilitates *π–π* stacking interactions, driving J‐aggregation within the interlayer (Figure 1h). The lateral separation between two Cu atoms in adjacent layers of CuNiPc is approximately 5.3 Å. Such a stacking mode of CuNiPc is further evidenced by the UV–vis absorption spectra (Figure S16).

In addition, the chemical states and local coordination environments of the central metal sites in CuNiPc/BiVO_4_ were analyzed by X‐ray absorption near‐edge structure (XANES) and extended X‐ray absorption fine structure (EXAFS) spectra. Figure 1i displays the Cu K‐edge XANES spectra of CuNiPc/BiVO_4_, CuCuPc/BiVO_4_, and CuPc/BiVO_4_, with Cu foil, CuPc, and Cu_2_O as references. The CuNiPc/BiVO_4_, CuCuPc/BiVO_4_ and CuPc/BiVO_4_ exhibit strong peaks located at around 8981 eV, which are assigned to the 1s→4p transition of the square planar Cu–N_4_ moiety [33]. The Cu K‐edge spectra reveal that the absorption edge of CuNiPc/BiVO_4_ locates between those of copper (II) phthalocyanine (CuPc) and cuprous oxide (Cu_2_O), indicating that the average valence state of Cu over CuNiPc is between + 1 and + 2 in the nanocomposite [34]. Notably, the near‐edge absorption energy of Cu in CuNiPc/BiVO_4_ negatively shifts in comparison to those of CuCuPc/BiVO_4_ and CuPc/BiVO_4_, indicating a lower valence state of Cu in CuNiPc, which is consistent with XPS results (Figure S7). In parallel, as depicted in Ni K‐edge XANES spectra (Figure 1k), the absorption edge of Ni over CuNiPc/BiVO_4_ locates between those of NiPc and Ni foil, and closer to NiPc [15]. Furthermore, according to the Fourier transform extended X‐ray absorption fine structure (FT‐EXAFS) spectra, both the Cu and Ni K‐edges in CuNiPc/BiVO_4_ show a prominent peak at around 1.50 Å, corresponding to the Cu–N and Ni–N scattering path, respectively (Figure 1j,l; Table S1) [33]. Besides, the FT‐EXAFS fitting curves in R space of the CuNiPc/BiVO_4_ (Figure S17a,b and Table S1) show that the coordination numbers of four fit the intrinsic Cu–N_4_ and Ni–N_4_ structure. To further clarify the coordination conditions of Cu and Ni atoms over CuNiPc/BiVO_4_, wavelet transform EXAFS (WT‐EXAFS) was performed (Figure S17c,d). Specifically, the intensity maximum at 4.5 and 5.2 Å^−1^ for Cu and Ni are close to the value for the CuPc and NiPc [33], which is attributed to the Ni–N or Cu–N scattering paths in the first coordination shell. Notably, the absence of characteristic peaks assigned to Cu–Cu (2.27 Å) and Ni–Ni bonds (2.18 Å) exclude the Cu/Ni clusters or crystalline particles in the CuNiPc/BiVO_4_ [35].

### 2.2 Photocatalytic Performance and Charge Separation

**FIGURE 2:**
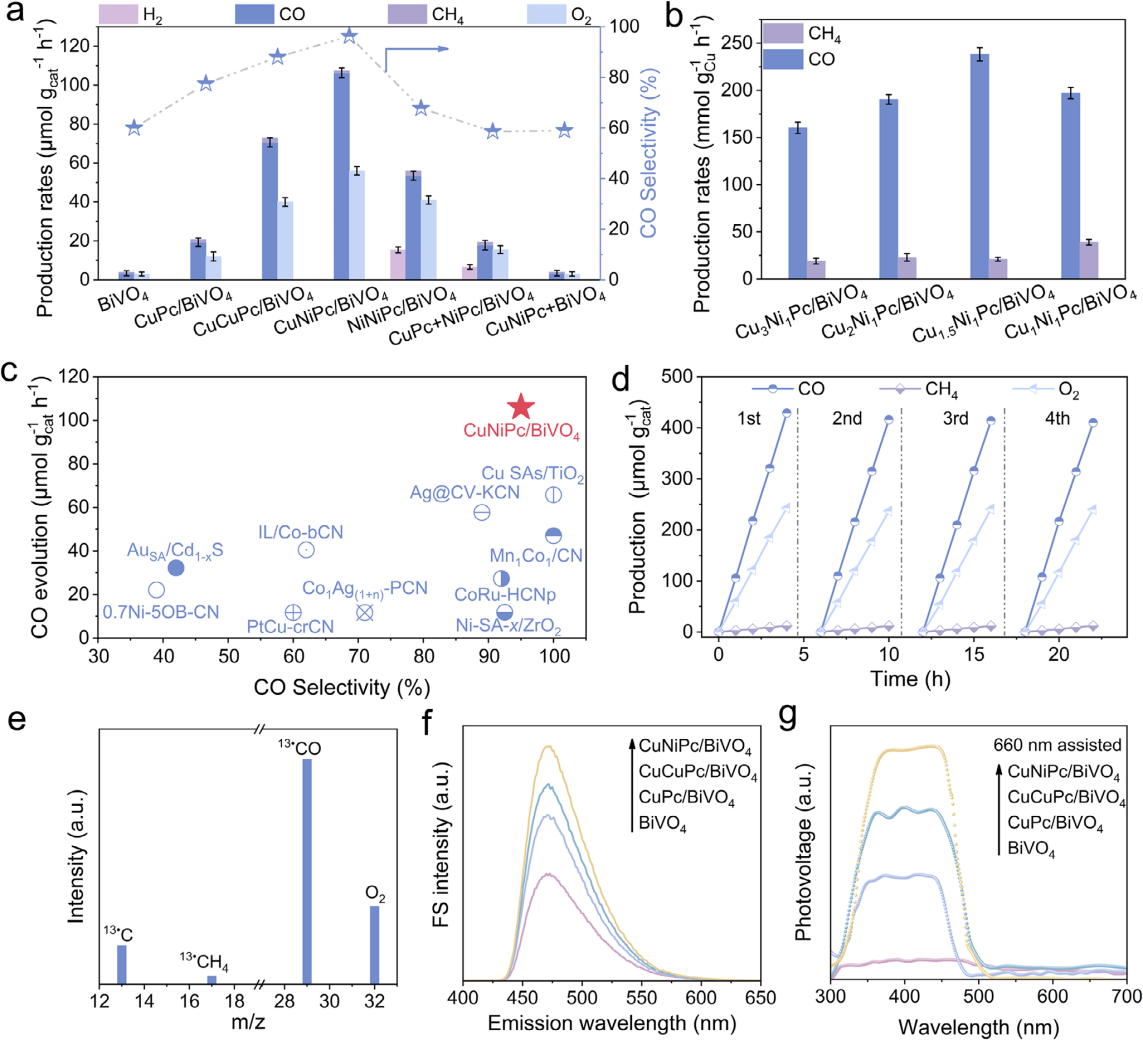
(a) Photocatalytic activities for CO_2_ reduction of the investigated catalysts. (b) Photocatalytic CO_2_ reduction performance of CuNiPc/BiVO_4_ with varied molar ratio of Cu to Ni under light irradiation. (c) Comparison of CO evolution rates and selectivity for recently reported photocatalysts and CuNiPc/BiVO_4_. (d) Photocatalytic cycling test of CuNiPc/BiVO_4_ heterojunction. (e) Mass spectra of the products from the photocatalytic reduction of ^13^CO_2_ over CuNiPc/BiVO_4_. (f) FS spectra related to the formed •OH amounts and (g) SPS responses of CuNiPc/BiVO_4_, CuCuPc/BiVO_4_, CuPc/BiVO_4_，and BiVO_4_ assisted with a 660 nm monochromatic beam, respectively.

The photocatalytic CO_2_ reduction performance of the investigated samples was evaluated in pure water (Figure 2a). CO along with a certain amount of CH_4_ and H_2_ were detected as the reduction products on the catalysts, and no other gaseous product and liquid products were detected under the current experimental conditions. One can see the CO yield of BiVO_4_ is improved after CuPc modification. While CuCuPc/BiVO_4_ one delivers superior activity in comparison to CuPc/BiVO_4_, achieving a CO production rate of 70.6 µmol h^−1^ g^−1^ with a selectivity of 88.0%. In contrast, NiNiPc/BiVO_4_ shows an inferior CO evolution rate (53.5 µmol h^−1^ g^−1^) and a low selectivity of 67.8% with H_2_ (15.4 µmol h^−1^ g^−1^) as an undesired by‐product. A similar trend is observed on NiPc/BiVO_4_ in comparison to mononuclear CuPc/BiVO_4_ (Figure S18). This result indicates that the selectively of CO_2_ reduction is closely related to the catalogue of metal active sites and the inherent structures of MPc. Remarkably, the CuNiPc/BiVO_4_ heterojunction demonstrates superior photocatalytic performance, achieving a CO production rate of 106.3 µmol h^−1^ g^−1^ with 96.3% selectivity, noteworthily without any H_2_ evolution. Noticeably, a certain amount of O_2_ was also detected on the investigated samples as the oxidant products in the overall CO_2_ reduction reaction. Moreover, a control experiment with physically mixed CuNiPc/BiVO_4_ by grinding the as‐prepared CuNiPc and BiVO_4_ together exhibits a quite low CO production rate, and the mixture of CuPc+NiPc/BiVO_4_ hybrid also yields a moderate CO production rate, yet still substantially lower than the CuNiPc/BiVO_4_. In parallel, H_2_ was still evolving. This observation highlights the synergistic effect of molecular‐level metal coordination over CuNiPc, which could improve CO_2_ reduction selectivity and inhibit H_2_ evolution.

In addition, the photocatalytic performance of CuNiPc/BiVO_4_ nanocomposites with varied molar ratios of Cu to Ni was evaluated, in which the Cu_1.5_Ni_1_Pc/BiVO_4_ exhibits the optimal CO production of 238 mmol g_Cu_^−1^ h^−1^ (Figure 2b). The contents of Cu and Ni in Cu_1.5_Ni_1_Pc are determined to be 4.46 and 4.12 wt.%, respectively (Table S2). When the CuNiPc loadings are varied from 0.5 to 1.5 wt.%, the measured CO yields present a volcanic trend, and 1.0 wt.% CuNiPc/BiVO_4_ shows the best photocatalytic CO_2_ reduction performance (Figure S19).

Furthermore, other heterobimetallic phthalocyanines (CuCoPc, CuMnPc, CuZnPc and CuFePc) modified BiVO_4_ were also prepared and evaluated the photoactivities (Figure S20). Comparative photocatalytic studies reveal that CuNiPc/BiVO_4_ maintains superior activity among all the catalysts, underscoring the preferable electronic coupling between Cu and Ni centers for CO_2_ reduction and charge separation. Notably, the CO yield and selectivity of CuNiPc/BiVO_4_ rank among the forefront of the single‐atom supported catalysts for photocatalytic CO_2_ reduction (Figure 2c; Table S3). The stability and recyclability of the catalyst wer evaluated with CuNiPc/BiVO_4_ as a representative (Figure 2d). The CO and CH_4_ yields gradually increase along with illumination time, and the detected reduction and oxidative products have no noteworthy decrease during the consecutive four cycles. Then, a ^13^CO_2_ isotope labeling experiment was carried out to validate the carbon source of the photocatalytic product. The mass fragments corresponding to ^13^CO (m/z = 29) and ^13^CH_4_ (m/z = 17) are detected when using ^13^CO_2_ as the feedstock under identical reaction conditions (Figure 2e). The above results confirm that the CO product indeed comes from photocatalytic CO_2_ reduction rather than the decomposition of CuNiPc under light irradiation. Meanwhile, the structural features of CuNiPc/BiVO_4_ are well retained after the reaction as revealed by DRS spectra, FT‐IR spectra, and XPS analyses (Figures S21–S23). In parallel, no obvious aggregation of CuNiPc is observed by TEM image (Figure S24). The above result suggests that the prepared catalyst delivers good stability.

The charge separation properties of the as‐fabricated catalysts were evaluated with fluorescence (FS) spectra related to the formed hydroxyl radical amounts (Figure 2f). The amounts of formed hydroxyl radicals are quantified by adding the probe molecule coumarin to generate the luminescent 7‐hydroxy coumarin molecules. In comparison with pristine BiVO_4_, all the metal phthalocyanines modified ones show much stronger FS intensities, and the highest FS signal is observed on CuNiPc/BiVO_4_, implying the best charge separation. The behavior of the photogenerated charge carrier was then explored by the steady‐state surface photovoltage spectroscopy (SS‐SPS), a typical photophysical technique that could reveal the charge separation and recombination by means of the surface potential difference of a semiconductor before and after illumination. In order to rule out the influence of oxygen trapping electrons, the SPS measurement was conducted in a N_2_ atmosphere. An additional 660 nm monochromatic excitation beam was introduced to guarantee the simultaneous excitation of BiVO_4_ and metal phthalocyanines (Figure 2g). A negligible SPS signal is recorded on BiVO_4_ due to the rapid recombination of photogenerated electrons and holes. However, a pronounced SPS response is observed on CuNiPc/BiVO_4_, demonstrating significantly enhanced charge separation. Interestingly, the CuNiPc/BiVO_4_ generates a stronger photovoltage than its homonuclear and mononuclear counterparts (CuCuPc/BiVO_4_ and CuPc/BiVO_4_), suggesting superior charge transport properties. In addition, the photocurrent response and electrochemical impedance spectroscopy (EIS) were employed to investigate the charge transfer and separation of samples. Phthalocyanine‐modified BiVO_4_ exhibited significantly enhanced photocurrent compared with that of BiVO_4_. The CuNiPc/BiVO_4_ delivers the strongest photocurrent (Figure S25a). Simultaneously, the EIS spectra of CuNiPc/BiVO_4_ (Figure S25b) also demonstrate the smallest semicircle, suggesting the best charge separation. The electrochemical results are in line with the SPS responses and FS measurement.

### 2.3 Confirmation of Z‐Scheme Charge Transfer

**FIGURE 3:**
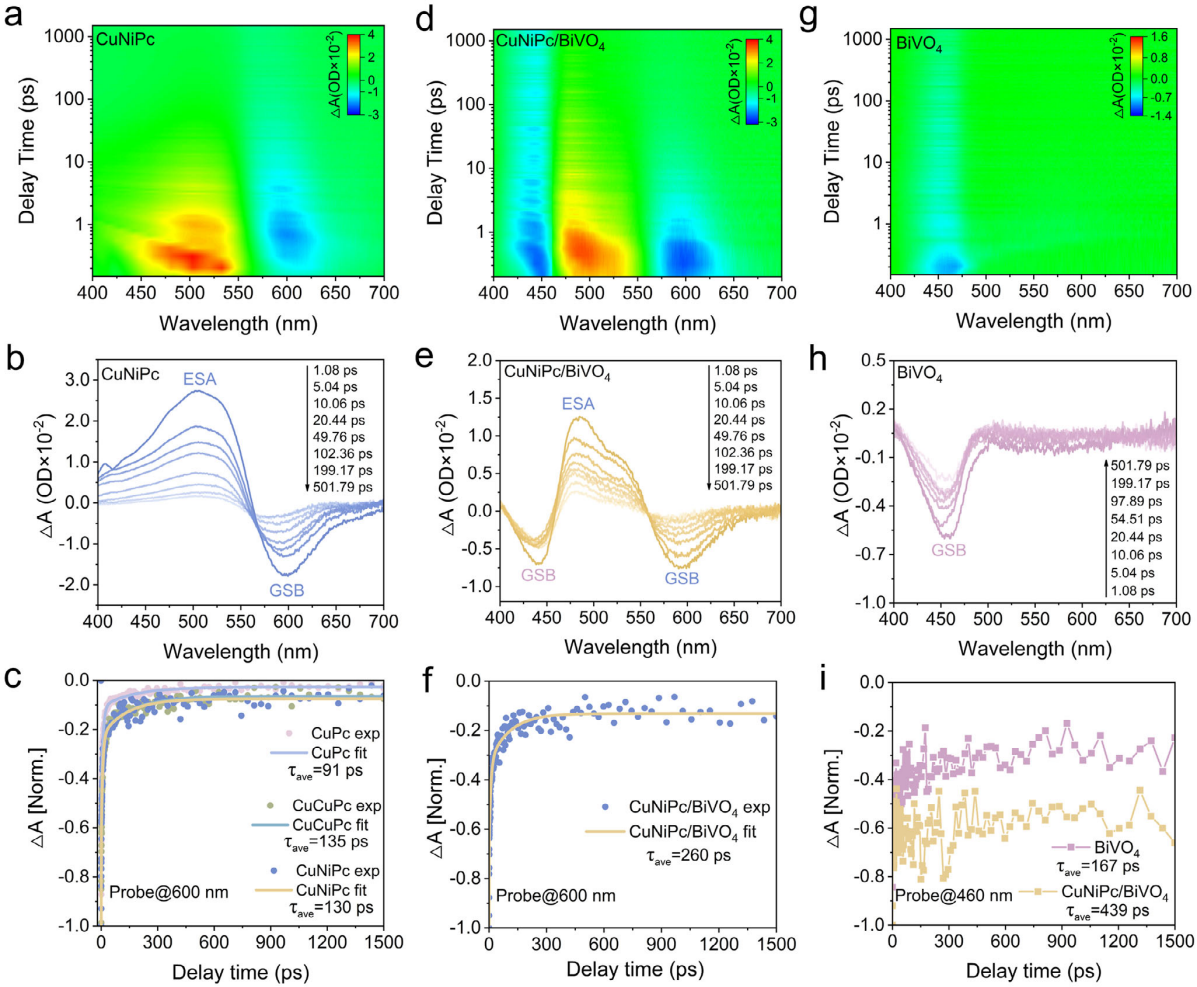
Femtosecond transient absorption (fs‐TA) contour maps and spectra at various delay times for (a,b) CuNiPc, (d,e) CuNiPc/BiVO_4_, and (g,h) BiVO_4_ following 355 nm laser excitation. The corresponding kinetic decay traces of characteristic signals at (c,f) 600 nm and (i) 460 nm, respectively.

To explore the charge transfer manner between BiVO_4_ and CuNiPc and elucidate the photoexcited carrier dynamics, femtosecond time‐resolved transient absorption spectroscopy (fs‐TAS) was performed. The experiments employed 355 nm pump pulses for photoexcitation, with subsequent probe pulses monitoring the spectral evolution of excited states. The photoexcited carrier dynamics of CuNiPc, CuCuPc and CuPc were characterized by 2D fs‐TA mapping and decay kinetics over 1–1500 ps delays (Figure 3a,b; Figure S26). The positive signal in the range of 450–550 nm is attributed to the excited‐state absorption (ESA), while the negative signal in the range of 570–650 nm is assigned to the ground‐state bleaching (GSB) of phthalocyanines [7]. The GSB lifetime reflects the charge kinetic decay process, which is in line with charge separation and transfer conditions [36]. Notably, CuNiPc and CuCuPc exhibit enhanced GSB signals compared to CuPc, demonstrating more efficient ground‐state electron promotion to excited states [37]. Decay kinetics are analyzed using tri‐exponential fitting at 600 nm, CuNiPc and CuCuPc exhibit similar but long‐lived ground‐state bleaching decay (130 and 135 ps, respectively) than CuPc (91 ps) (Figure 3c; Table S4). This trend has also been observed in the nickel‐based catalysts (Figure S27 and Table S5), where NiNiPc exhibits an extended average lifetime (115 ps) relative to NiPc (81 ps). Furthermore, CuNiPc presents the strongest intramolecular electric field (IEF) value, which is 1.6 and 3.1 times higher than those of CuCuPc and CuPc (Figure S28), respectively. These indicate that binuclear phthalocyanines are more favorable for the charge separation compared to their mononuclear counterparts. The fs‐TA spectra of CuNiPc/BiVO_4_ reveal not only the characteristic GSB and ESA features of CuNiPc but also an additional negative band (400–475 nm), attributed to the GSB signal of BiVO_4_ due to electron accumulation in its conduction band (Figure 3d,e) [38]. This assignment is corroborated by the identical spectral feature observed in pristine BiVO_4_ (Figure 3g,h). The charge‐separated state of CuNiPc in the CuNiPc/BiVO_4_ heterojunction gradually evolved into the ground state with a long decay time of 260 ps (Figure 3f; Table S6).

It is noteworthy that the decay of CuNiPc in the CuNiPc/BiVO_4_ heterojunction at 600 nm is nearly two times longer than that of the pristine CuNiPc. This pronounced extension of carrier lifetime unambiguously confirms the Z‐scheme charge transfer route, which effectively sustains long‐lived charge carriers for photocatalysis. Moreover, transient absorption spectroscopy at 460 nm reveals a 2.6‐fold enhancement in charge carrier lifetime for BiVO_4_ within the heterojunction (439 ps) compared to pristine BiVO_4_ (167 ps) (Figure 3i; Table S7), giving direct experimental evidence of significantly suppressed electron‐hole recombination at the interface. These results observed across both components provide definitive spectroscopic validation of the Z‐scheme charge transfer manner in the heterojunction.

**FIGURE 4:**
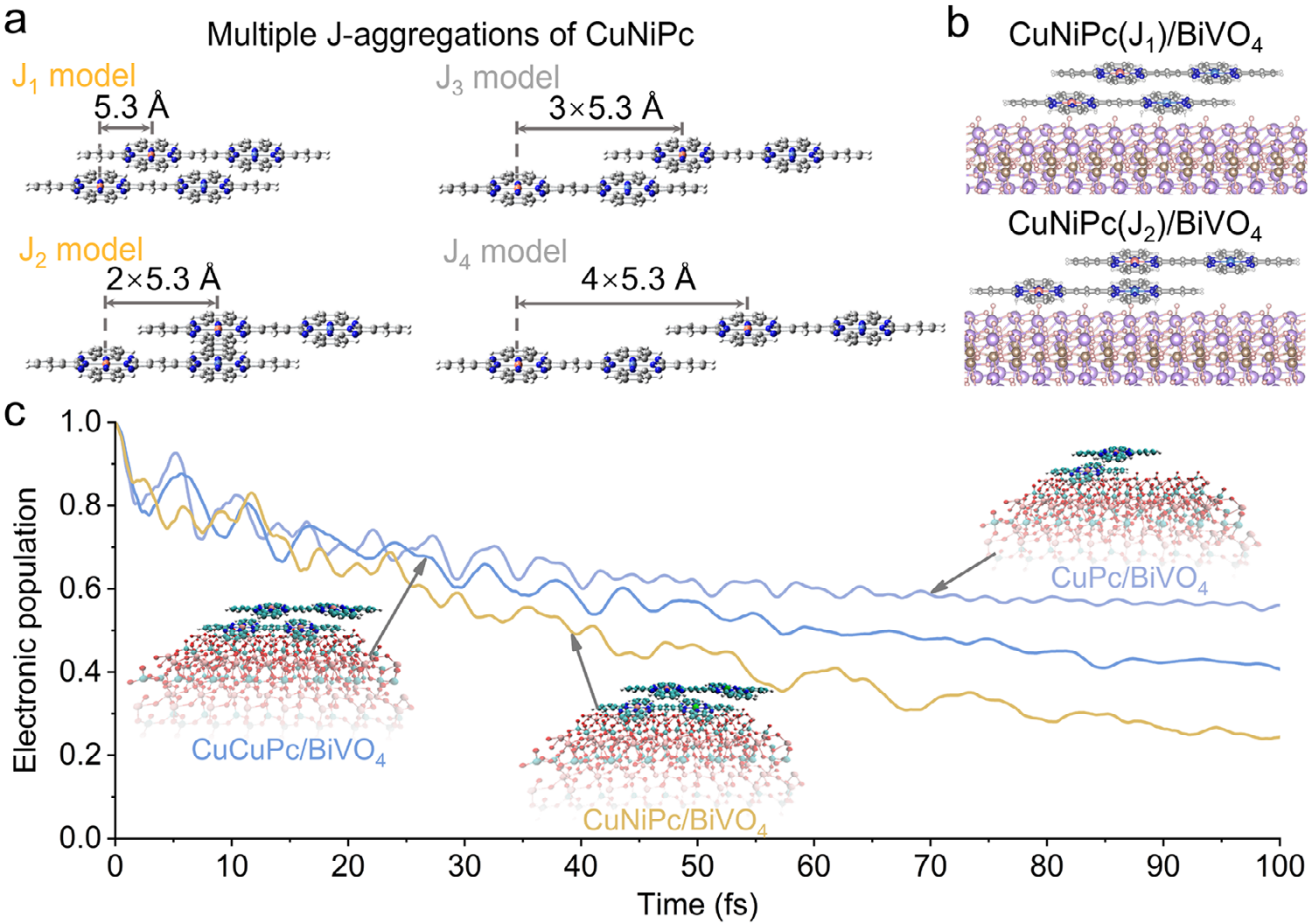
(a) Side view of different dimer configurations of the CuNiPc J‐aggregates. (b) Side view of the DFT optimized CuNiPc(J_1_, J_2_)/BiVO_4_ heterojunction. (c) The time‐dependent survival probability (TDSP) curves of the excited electrons transfer from BiVO_4_ to CuNiPc, CuCuPc, and CuPc during the injection process.

Moreover, the interfacial electron transfer processes of CuNiPc/BiVO_4_, CuCuPc/BiVO_4_, and CuPc/BiVO_4_ under excited‐state were further simulated by density functional theory (DFT) calculations. It is well known that phthalocyanine molecules with planar structure could form stacked aggregates through *π–π* interaction. The CuNiPc dimer with different conformations are considered to simulate the J‐aggregation properties of CuNiPc. Four molecular stacking configurations are constructed based on the displacement difference of the stable conformations for the CuNiPc dimer, namely J_1_, J_2_, J_3_, and J_4_‐models, respectively (Figure 4a; Figure S29). One can see the molecules still have the planar structure, but an obvious displacement between the two planes. The displacements of two CuNiPc molecules are 5.3 Å and 10.6 Å in the J_1_‐model and J_2_‐model, respectively, while there is less overlap of the electron cloud density between the molecules due to larger displacements in the J_3_‐model and J_4_‐model. Thus, the conformation of the J_3_‐model and J_4_‐model can be neglected in subsequent calculations. Similarly, the conformation of the J_1_‐model of CuPc aggregates is reserved (Figure S30).

Subsequently, to deeply investigate the charge transfer mechanism at the phthalocyanine/BiVO_4_ interface, the models of CuNiPc(J_1_, J_2_)/BiVO_4_, CuCuPc(J_1_, J_2_)/BiVO_4_, and CuPc(J_1_)/BiVO_4_ heterojunctions are constructed (Figure 4b; Figure S31).

Then, the excited‐state interfacial electron transfer process from BiVO_4_ to phthalocyanines, namely Z‐scheme charge transfer at the interface, is calculated (Figure 4c). The kinetic results of the excited‐state interfacial electron transfer show that 50% of the interfacial electrons in the CuPc/BiVO_4_ system are transferred within 100 fs. However, for the CuCuPc modified one, the interfacial electron transfer is significantly promoted, and its electron transfer half‐life is shortened to 57 fs. Notably, much faster interfacial charge injection is observed on CuNiPc/BiVO_4_, where 50% of the electron transfer is achieved in only 37 fs, indicating this process is further accelerated with the synergy of Cu and Ni sites in CuNiPc. These results support that the interfacial Z‐scheme charge transfer is more liable to be regulated by heteronuclear phthalocyanine CuNiPc. The theoretical simulations are in line with the fs‐TAS results.

**FIGURE 5:**
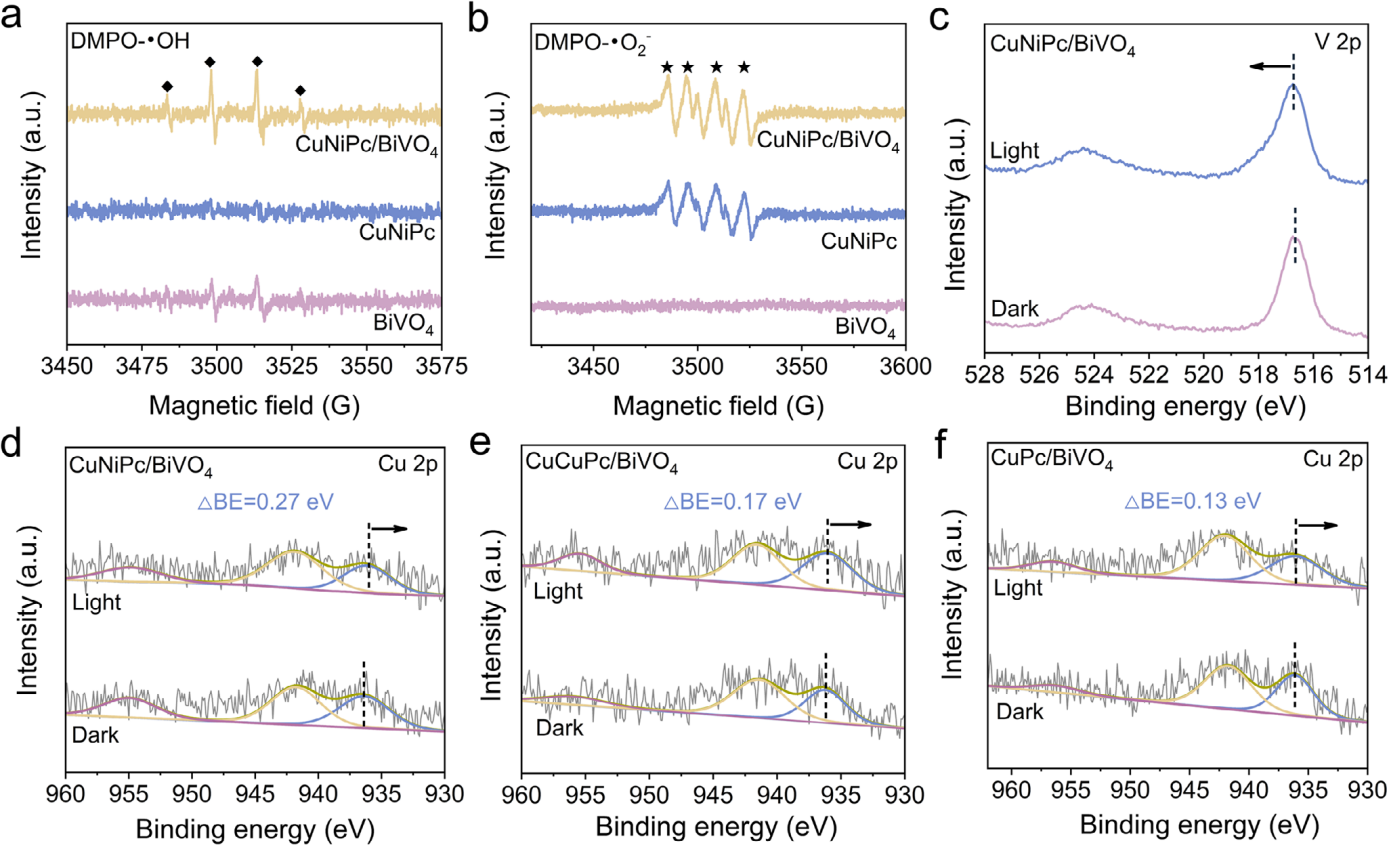
DMPO spin‐trapping EPR spectra recorded for (a) •OH and (b) •O_2_^−^ of CuNiPc, CuNiPc/BiVO_4_, and BiVO_4_ under light irradiation. In situ irradiated XPS analyses for (c) V 2p of CuNiPc/BiVO_4_, Cu 2p of (d) CuNiPc/BiVO_4_, (e) CuCuPc/BiVO_4_ and (f) CuPc/BiVO_4_ before and after light irradiation.

The electron paramagnetic resonance (EPR) spectra with 5, 5‐dimethyl‐1‐pyrroline N‐oxide (DMPO) as the spin‐electron trapping agents were further support the above results (Figure 5a,b). An obvious DMPO‐•OH EPR signal is recorded on BiVO_4_, yet no detectable signal is observed for CuNiPc, which is closely related to the energy band structures of the photocatalysts (Figures S32 and S33). However, a much higher EPR signal is obtained on CuNiPc/BiVO_4_, indicating that more photogenerated holes accumulated on BiVO_4_ to produce •OH radicals. On the other hand, CuNiPc presents a DMPO‐•O_2_^−^ EPR signal under light irradiation, in contrast, a silence EPR signal is detected on BiVO_4_. While CuNiPc/BiVO_4_ one exhibits a much stronger EPR response than that of CuNiPc. The above results suggest that the charge transfer between CuNiPc and BiVO_4_ obeys the Z‐scheme pathway. In situ irradiated XPS was then performed to explore the charge transfer direction [39]. During light irradiation, a positive shift of Bi 4f and V 2p peaks on CuNiPc/BiVO_4_ is clearly observed compared to those in the dark (Figure 5c; Figure S34), while an opposite shift direction is detected for N 1s, Cu 2p, and Ni 2p (Figure 5d; Figures S35 and S36). Such a BE variation indicates an accumulation of electrons within CuNiPc under illumination, consistent with charge transfer across the CuNiPc/BiVO_4_ interface following a Z‐scheme pathway. Moreover, it is noticed that the BE variation of Cu 2p over CuNiPc/BiVO_4_ is more pronounced than that of Ni 2p, hinting that the Cu–N_4_ unit of CuNiPc potentially be the active sites for CO_2_ photoreduction. Interestingly, the BE variation of Cu 2p on CuNiPc/BiVO_4_ (0.27 eV) under light irradiation is more pronounced than those recorded on CuCuPc/BiVO_4_ (0.17 eV) and CuPc/BiVO_4_ (0.13 eV), respectively (Figure 5d–f). This is possibly due to the preferable conductivity and higher carrier density of the heteronuclear CuNiPc (Figure S37 and Table S8).

### 2.4 Mechanism of CO_2_ Conversion

The CO_2_ adsorption properties of the investigated catalysts were revealed by CO_2_ adsorption isotherms (Figure S38). One can notice that the CO_2_ adsorption is greatly promoted after phthalocyanines modification, implying the introduced phthalocyanine provide the adsorption sites. The CuNiPc/BiVO_4_ exhibits stronger CO_2_ adsorption capacity than CuCuPc/BiVO_4_ and CuPc/BiVO_4_ ones. Besides, as demonstrated by electrochemical reduction curves (Figure S39), the onset potential of CuPc/BiVO_4_ is much lower than pristine BiVO_4_, and it further decreased on CuCuPc modified one. Remarkably, CuNiPc/BiVO_4_ delivers the lowest onset potential, suggesting that it is more prone to CO_2_ activation on CuNiPc.

**FIGURE 6:**
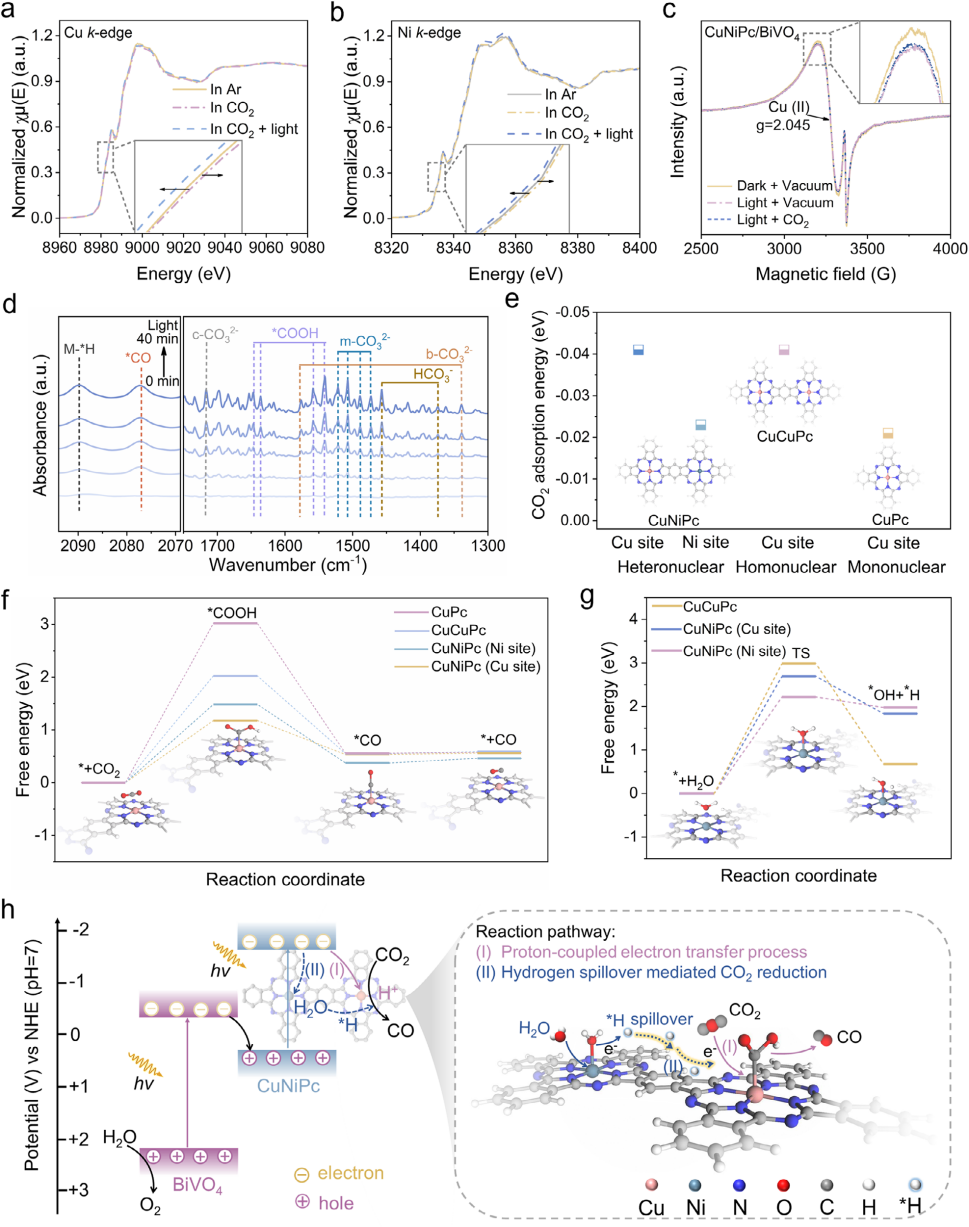
(a,b) Cu and Ni K‐edge XANES spectra of CuNiPc/BiVO_4_ under conditions of Ar, CO_2_ and CO_2_ with illumination. (c) In situ EPR spectra of CuNiPc/BiVO_4_. (d) In situ DRIFTS for the detected intermediates of CuNiPc/BiVO_4_. (e) CO_2_ adsorption energies on metal sites of CuNiPc, CuCuPc and CuPc. (f) Free energy diagram for CO_2_ photoreduction to CO on metal atoms over CuNiPc, CuCuPc and CuPc. (g) Free energy diagram for H_2_O dissociation on metal atoms over CuNiPc and CuCuPc. (h) Schematic of the involved charge transfer and redox reactions on CuNiPc/BiVO_4_ and the proposed reaction pathway of photocatalytic CO_2_ reduction over CuNiPc.

To gain deep insight into the specific role of Cu and Ni sites in CuNiPc/BiVO_4_ for photocatalytic CO_2_ reduction, in situ XAFS measurements were performed to monitor the electronic state of metal sites under CO_2_RR operating conditions. The Cu K‐edge of CuNiPc/BiVO_4_ shifts toward higher energy in the presence of CO_2_ compared to that in Ar atmosphere (Figure 6a), implying the loss of electrons from Cu and hence an increase in the oxidation state of Cu. Such an observation may be attributed to interaction between the metal sites and the adsorbed CO_2_ [40]. Under light irradiation, the Cu K‐edge of CuNiPc/BiVO_4_ moves toward lower energy, indicating that photogenerated electrons are transferred to Cu, gradually restoring the oxidation state of the Cu. For the Ni K‐edge XANES spectra of CuNiPc/BiVO_4_ (Figure 6b), a similar trend of change is observed under conditions of Ar, CO_2_ and CO_2_ with illumination, yet the variation on the Ni site is relatively small. As a result, it can be speculated that Cu–N_4_ moiety in the CuNiPc is serving as the dominant active site for CO_2_ adsorption and conversion.

Following the indication of in situ XAFS measurements, in situ EPR measurements were conducted to unveil the charge transfer behavior and active sites during the photocatalytic CO_2_ reaction over CuNiPc/BiVO_4_ (Figure 6c). A characteristic EPR signal with a g factor of 2.045 is detected in the ldark, which is assigned to Cu (II) [41], while the Ni signal is shielded by the strong Cu signal, such a phenomenon has also been observed in other studies [42]. Under light irradiation, the EPR signal of Cu (II) weakens compared with that in the dark, indicating the electrons are captured by Cu (II) and form Cu (I) with EPR silence. When CO_2_ is introduced into the system, the Cu (II) signal is partially recovered, suggesting that the electrons transfer from the Cu site to CO_2_ for triggering the reduction reaction.

In situ diffuse reflectance infrared Fourier transform spectroscopy (DRIFTS) was carried out to monitor the adsorption behavior of reactant and the produced intermediates during the photocatalytic CO_2_ conversion process. The peaks in the range of 2280–2400 cm^−1^ corresponding to the asymmetric stretching vibration of O═C═O for CO_2_ [43] is detected on all the investigated catalysts (Figure S40). The CuNiPc/BiVO_4_ and CuCuPc/BiVO_4_ display much stronger CO_2_ adsorption peaks than those of CuPc/BiVO_4_ and pristine BiVO_4_ in the dark, which is in line with the CO_2_ adsorption isotherms result. It is worth noting that a signal (3000–3800 cm^−1^) attributed to the O─H stretching vibrations of water is recorded on CuNiPc/BiVO_4_ (Figure S41). Specifically, the fitted peaks locate at high wavenumber (> 3500 cm^−1^) is considered to be H_2_O with a weakly H‐bonded structure, which is more likely to be adsorbed on surface of the catalyst to undergo cleavage and produce active hydrogen [44, 45]. The content of H_2_O with a weakly H‐bonded structure in CuNiPc/BiVO_4_ reaches 21%, while that of CuCuPc/BiVO_4_ is only 16%. Upon light irradiation, the peak (at 2089 cm^−1^) assigned to the ^*^H bonded on the metal surface [35, 46] over CuNiPc/BiVO_4_ is detected (Figure 6d). In contrast, it can be hardly observed such signals over CuCuPc/BiVO_4_ and CuPc/BiVO_4_ (Figure S42). The above results suggest that the incorporation of Ni–N_4_ moiety in CuNiPc/BiVO_4_ facilitates the H_2_O adsorption/activation and the formation of active ^*^H.

In parallel, the asymmetric stretching vibration of O═C═O on heterojunctions is significantly weakened, whereas the change for CuNiPc/BiVO_4_ is the most pronounced among the investigated samples. Typical carbonate species associated with monodentate carbonates (m‐CO_3_^2−^: 1520, 1508, 1488, and 1473 cm^−1^) [47, 48], bidentate carbonates (b‐CO_3_^2−^: 1577 and 1338 cm^−1^) [49], chelating bridged carbonate (c‐CO_3_^2−^: 1715 cm^−1^) [50] and ^*^HCO_3_^−^ (1456 and 1373 cm^−1^) [47] are observed (Figure 6d; Figures S42 and S43). More importantly, the distinctive signal of ^*^COOH species (at 1646, 1635, 1560, and 1540 cm^−1^) [48, 49], a key intermediate involved in CO_2_‐to‐CO conversion, increasing under prolonged illumination (Figure 6d; Figures S42 and S43). The CuNiPc/BiVO_4_ shows the strongest signal of ^*^COOH species. In addition, the absorption peaks assigned to ^*^CO at 2077 cm^−1^ appears and increase under illumination [51], indicating efficient CO_2_ conversion on the catalysts. The normalized relative peak intensities (Figure S44) evidence that the CuNiPc/BiVO_4_ shows the fastest increase in ^*^CO production among CuNiPc/BiVO_4_, CuCuPc/BiVO_4_, and CuPc/BiVO_4_, hinting the preferable CO_2_ reduction process. This is closely related to the Ni–N_4_ sites, which facilitate the H_2_O activation to generate ^*^H, which subsequently undergoes intramolecular spillover to the Cu–N_4_ site, accelerating the ^*^COOH generation in photocatalytic CO_2_ reduction.

Moreover, the DFT calculations were performed to investigate the synergy of Cu and Ni sites in CuNiPc. The CO_2_ adsorption energies on the Cu sites of CuPc and CuCuPc are calculated to be ‐0.021 and ‐0.041 eV, respectively (Figure 6e; Figure S45), demonstrating the binuclear phthalocyanine is more favorable for the CO_2_ adsorption. Remarkably, the CO_2_ adsorption energy on the Cu site of CuNiPc is larger than that on the Ni site, suggesting the stronger binding between Cu–N_4_ and CO_2_. In addition, charge density difference analysis reveals preferential electron donation to ^*^CO_2_ from Cu sites (0.039 e) versus Ni sites (0.023 e) (Figure S46). Accordingly, it is suggested that Cu serves as the primary active center for CO_2_ adsorption and activation. Thereafter, the energy profile of the CO_2_ conversion to the CO pathway on the specific active sites of the investigated MPcs was conducted. Figure 6f shows the free energy profiles for CO_2_RR pathways at the Cu and Ni sites in CuNiPc, as well as at the Cu sites in CuCuPc and CuPc. Compared to CuPc (3.02 eV) and CuCuPc (2.03 eV), the rate‐determining step (RDS) ^*^CO_2_ ‐to‐^*^COOH on CuNiPc (1.18 eV) is thermodynamically favorable, and suggests the incorporation of Ni in CuNiPc facilitates the protonation process. Notably, the lower CO binding energy on the Cu sites of CuNiPc (0.56 eV) facilitates CO desorption compared to that on CuCuPc (0.59 eV) and CuPc (0.58 eV). In parallel, the H_2_O activation on CuCuPc and CuNiPc has also been simulated. As shown in Figure 6g, the dissociation of H_2_O into ^*^H and ^*^OH on phthalocyanines is an endothermic reaction. The energies for H_2_O activation at Cu sites in CuNiPc and CuCuPc are 2.69 and 2.99 eV, respectively, which may be related to the different electronic structure of Cu sites. Notably, less energy is required to dissociate H_2_O at the Ni site (2.23 eV) than at the Cu site in CuNiPc, suggesting that the incorporated Ni–N_4_ moiety in CuNiPc is favorable for H_2_O dissociation. Subsequently, the produced ^*^OH and ^*^H species dissociated by H_2_O are adsorbed at the Ni site and the adjacent N site, respectively. As illustrated in Figure S47a, the energy barrier of ^*^H conversion to H_2_ is much higher (1.68 eV) than that of ^*^COOH formation (1.18 eV) at the Cu site (Figure 6f), indicating that the H^*^ generated on the Ni–N_4_ moiety preferentially undergo spillover to the Cu site, where they promote ^*^COOH formation rather than H_2_ evolution. In contrast, the NiNiPc catalyst exhibits a significantly lower energy barrier for the hydrogen evolution reaction (HER, 0.63 eV) than for CO_2_ reduction (2.51 eV, Figure S47b), thermodynamically favoring H_2_ generation. This finding aligns well with the observed photocatalytic CO_2_ reduction performance and product selectivity on these investigated catalysts.

Based on the experimental observations and theoretical simulations, a plausible mechanism of charge transfer and separation as well as the subsequent CO_2_ photoreduction on CuNiPc/BiVO_4_ heterojunction is proposed (Figure 6h). Under light irradiation, photogenerated electrons of BiVO_4_ recombine with photoinduced holes of CuNiPc, and the remaining photoholes of BiVO_4_ oxidize H_2_O to produce O_2_, while the accumulated electrons of CuNiPc initiate CO_2_ conversion. In this procedure, the energetic electrons of CuNiPc transfer to Cu–N_4_ sites for CO_2_ adsorption and activation, and Ni–N_4_ sites facilitate H_2_O dissociation to supply protons (*H). Sequential reduction proceeds via ^*^COOH → ^*^CO → CO pathway. Crucially, the synergy of the Cu–N_4_ and Ni–N_4_ sites lower the energy barrier for rate‐determining ^*^COOH formation, accounting for the enhanced CO_2_ reduction performance.

## 3 Conclusion

In summary, heteronuclear dual‐metal phthalocyanine assemblies with atomically active sites have been successfully designed and integrated on BiVO_4_ nanosheets for selective CO_2_ photoreduction. The optimal CuNiPc/BiVO_4_ exhibits a CO evolution rate of 238 mmol g_Cu_^−1^ h^−1^ with nearly 100% CO selectivity under light irradiation. Such a novel CuNiPc architecture with the synergy of Cu and Ni atomic sites enables both CO_2_ activation and H_2_O dissociation to generate ^*^H followed by spillover to the Cu sites, significantly promoting the selectivity of CO_2_ reduction and suppressing the evolution of H_2_. The mechanism of fast interfacial Z‐scheme charge transfer in the CuNiPc/BiVO_4_ is primarily revealed by fs‐TAS and TDSP curves, while the specific role of Cu and Ni sites in CO_2_ reduction are confirmed by in situ XAS and in situ EPR, etc. This work establishes a molecular‐level engineering strategy for tailoring metal phthalocyanine catalysts toward artificial photosynthesis. The desirable structure of heteronuclear dual‐metal phthalocyanine is expected to show broader applicability, with potential extensions to other fields of energy production like H_2_O_2_ synthesis and N_2_ reduction.

## Funding

The National Natural Science Foundation of China (no. U2102211, U23A20576, 22202064), National Key R&D Program of China (no. 2024YFF0506202), Outstanding Youth Science Foundation of Heilongjiang Province (no. YQ2023B008), Postdoctoral Science Foundation of Heilongjiang Province (no. LBHZ22034)

## Conflicts of Interest

The authors declare no conflict of interest.

## Supporting information




**Supporting Information**: advs73582‐sup‐0001‐SuppMat.pdf

## Data Availability

The data that support the findings of this study are available from the corresponding author upon reasonable request.

## References

[1] B. R. Xu , S. C. Luo , W. B. Hua , et al., “Constructing Atomic Tungsten‐Based Solid Frustrated‐Lewis‐Pair Sites With D‐p Interactions for Selective CO2 Photoreduction,” Journal of the American Chemical Society 147, no. 1 (2025): 200–210, 10.1021/jacs.4c08953. 39692537

[2] D. C. Zhong , Y. C. Wang , M. Wang , and T. B. Lu , “Precise Synthesis of Dual‐Atom Catalysts for Better Understanding the Enhanced Catalytic Performance and Synergistic Mechanism,” Accounts of Chemical Research 58, no. 9 (2025): 1379–1391, 10.1021/acs.accounts.4c00855. 40207527

[3] Y. Zhang , X. Y. Zhang , L. Jiao , Z. Meng , and H. L. Jiang , “Conductive Covalent Organic Frameworks of Polymetallophthalocyanines as a Tunable Platform for Electrocatalysis,” Journal of the American Chemical Society 145, no. 44 (2023): 24230–24239, 10.1021/jacs.3c08594. 37890005

[4] J. I. Bian , J. Feng , Z. Zhang , et al., “Dimension‐Matched Zinc Phthalocyanine/BiVO 4 Ultrathin Nanocomposites for CO 2 Reduction as Efficient Wide‐Visible‐Light‐Driven Photocatalysts via a Cascade Charge Transfer,” Angewandte Chemie International Edition 58, no. 32 (2019): 10873–10878, 10.1002/anie.201905274. 31199043

[5] Y. X. Ye , J. H. Pan , F. Y. Xie , et al., “Highly Efficient Photosynthesis of Hydrogen Peroxide in Ambient Conditions,” PANS 118, no. 16 (2021): 2103964118, 10.1073/pnas.2103964118. PMC8072241 33853952

[6] G. Li , Z. Lian , Z. Wan , et al., “Efficient Photothermal‐assisted Photocatalytic NO Removal on Molecular Cobalt Phthalocyanine/Bi2WO6 Z‐Scheme Heterojunctions by Promoting Charge Transfer and Oxygen Activation,” Applied Catalysis B: Environmental 317 (2022): 121787, 10.1016/j.apcatb.2022.121787.

[7] N. Y. Li , J. Wang , G. H. Zhao , et al., “Enabling CsPbBr3 Perovskites for Photocatalytic CO2 Methanation by Rationalizing a Z‐Scheme Heterojunction With Zinc Phthalocyanine,” ACS Materials Letters 6, no. 3 (2024): 999–1006, 10.1021/acsmaterialslett.3c01550.

[8] L. B. Li , K. Yuan , and Y. W. Chen , “Breaking the Scaling Relationship Limit: From Single‐Atom to Dual‐Atom Catalysts,” Accounts of Materials Research 3, no. 6 (2022): 584–596, 10.1021/accountsmr.1c00264.

[9] T. Ouyang , H.‐J. Wang , H.‐H. Huang , et al., “Dinuclear Metal Synergistic Catalysis Boosts Photochemical CO 2 ‐to‐CO Conversion,” Angewandte Chemie International Edition 57, no. 50 (2018): 16480–16485, 10.1002/anie.201811010. 30362217

[10] Y. Y. Wang , Y. Qu , B. H. Qu , et al., “Construction of Six‐Oxygen‐Coordinated Single Ni Sites on G‐C3N4 With Boron‐Oxo Species for Photocatalytic Water‐Activation‐Induced CO2 Reduction,” Advanced Materials 33, no. 48 (2021): 2105482, 10.1002/adma.202105482. 34569106

[11] P. G. Liu , Z. X. Huang , X. P. Gao , et al., “Synergy Between Palladium Single Atoms and Nanoparticles via Hydrogen Spillover for Enhancing CO2 Photoreduction to CH4,” Advanced Materials 34, no. 16 (2022): 2200057, 10.1002/adma.202200057. 35212057

[12] S. X. Yang , Y. H. Yu , X. J. Gao , Z. P. Zhang , and F. Wang , “Recent Advances in Electrocatalysis With Phthalocyanines,” Chem Soc Rev 2021 50, no. 23: 12985–13011, 10.1039/D0CS01605E. 34751683

[13] N. Kobayashi , “Dimers, Trimers and Oligomers of Phthalocyanines and Related Compounds,” Coordination Chemistry Reviews 227, no. 2 (2002): 129–152, 10.1016/S0010-8545(02)00010-3.

[14] X. J. Wang , Y. Liu , Y. Wang , et al., “Electrochemical and Spectroscopic Study of Homo‐ and Hetero‐Dimetallic Phthalocyanines as Catalysts for the Oxygen Reduction Reaction in Acidic Media,” ChemElectroChem 5, no. 22 (2018): 3478–3485, 10.1002/celc.201800977.

[15] Z. Y. Yang , R. Z. Chen , L. Zhang , Y. H. Li , and C. Z. Li , “Recent Progress in Nickel Single‐Atom Catalysts for the Electroreduction of CO2 to CO,” Industrial Chemistry & Materials 2, no. 4 (2024): 533–555, 10.1039/D3IM00109A.

[16] S. X. Yang , Y. H. Yu , M. L. Dou , Z. P. Zhang , L. M. Dai , and F. Wang , “Two‐Two‐Dimensional Conjugated Aromatic Networks as High‐Site‐Density and Single‐Atom Electrocatalysts for the Oxygen Reduction Reaction,” Angewandte Chemie International Edition 2019, 58, 14724–14730, 10.1002/anie.201908023. 31418496

[17] N. Kobayashi , H. Miwa , and V. N. Nemykin , “Adjacent versus Opposite Type Di‐Aromatic Ring‐Fused Phthalocyanine Derivatives: Synthesis, Spectroscopy, Electrochemistry, and Molecular Orbital Calculations,” Journal of the American Chemical Society 124, no. 27 (2002): 8007–8020, 10.1021/ja0123812. 12095345

[18] T. Wang , W. Huang , T. Sun , et al., “Two‐Dimensional Metal‐Polyphthalocyanine Conjugated Porous Frameworks as Promising Optical Limiting Materials,” ACS Applied Materials & Interfaces 12, no. 41 (2020): 46565–46570, 10.1021/acsami.0c13990. 32946214

[19] K. J. Chen , M. Q. Cao , Y. Y. Lin , et al., “Ligand Engineering in Nickel Phthalocyanine to Boost the Electrocatalytic Reduction of CO2,” Advanced Functional Materials 32, no. 10 (2022): 2111322, 10.1002/adfm.202111322.

[20] H. B. Yin , F. Dong , D. S. Wang , and J. H. Li , “Coupling Cu Single Atoms and Phase Junction for Photocatalytic CO2 Reduction With 100% CO Selectivity,” ACS Catalysis 12, no. 22 (2022): 14096–14105, 10.1021/acscatal.2c04563.

[21] J. Li , Q. S. Zhu , A. Chang , et al., “Molecular‐scale CO Spillover on a Dual‐site Electrocatalyst Enhances Methanol Production From CO2 Reduction,” Nature Nanotechnology 20, no. 4 (2025): 515–522, 10.1038/s41565-025-01866-8. 39966685

[22] Y. Y. Fan , W. C. Zhou , X. Qiu , et al., “Selective Photocatalytic Oxidation of Methane by Quantum‐Sized Bismuth Vanadate,” Nature Sustainability 4, no. 6 (2021): 509–515.

[23] C. Zhu , M. Z. Yang , B. Jiang et al., “Insights Into Excitonic Behavior in Single‐atom Covalent Organic Frameworks for Efficient Photo‐Fenton‐Like Pollutant Degradation,” Nature Communications 16, no. 1 (2025): 790, 10.1038/s41467-025-56103-6. PMC11742440 39824825

[24] M. Liu , N. Li , S. H. Cao , et al., “A “Pre‐Constrained Metal Twins” Strategy to Prepare Efficient Dual‐Metal‐Atom Catalysts for Cooperative Oxygen Electrocatalysis,” Advanced Materials 34, no. 7 (2022): 2107421, 10.1002/adma.202107421. 34862677

[25] S. Gao , B. Gu , X. Jiao , et al., “Highly Efficient and Exceptionally Durable CO2 Photoreduction to Methanol Over Freestanding Defective Single‐Unit‐Cell Bismuth Vanadate Layers,” Journal of the American Chemical Society 139, no. 9 (2017): 3438–3445, 10.1021/jacs.6b11263. 28208016

[26] Z. L. Zhao , J. Bian , L. N. Zhao , et al., “Construction of 2D Zn‐MOF/BiVO4 S‐scheme Heterojunction for Efficient Photocatalytic CO2 Conversion Under Visible Light Irradiation,” Chinese Journal of Catalysis 43, no. 5 (2022): 1331–1340, 10.1016/S1872-2067(21)64005-6.

[27] S. X. Yang , Y. H. Yu , M. L. Dou , Z. P. Zhang , and F. Wang , “Edge‐Functionalized Polyphthalocyanine Networks With High Oxygen Reduction Reaction Activity,” Journal of the American Chemical Society 142, no. 41 (2020): 17524–17530, 10.1021/jacs.0c07249. 32942851

[28] Y. Liu , H. S. Shang , B. Zhang , D. P. Yan , and X. Xiang , “Surface Fluorination of BiVO4 for the Photoelectrochemical Oxidation of Glycerol to Formic Acid,” Nature Communications 15, no. 1 (2024): 8155, 10.1038/s41467-024-52161-4. PMC11408720 39289360

[29] J. Chen , K. Zou , P. Ding , et al., “Conjugated Cobalt Polyphthalocyanine as the Elastic and Reprocessable Catalyst for Flexible Li–CO2 Batteries,” Advanced Materials 31, no. 2 (2019): 1805484, 10.1002/adma.201805484. 30393896

[30] G. De la Torre , G. Bottari , M. Sekita , A. Hausmann , D. M. Guldi , and T. Torres , “A Voyage Into the Synthesis and Photophysics of Homo‐ and Heterobinuclear Ensembles of Phthalocyanines and Porphyrins,” Chemical Society Reviews 42, no. 20 (2013): 8049–8105, 10.1039/C3CS60140D. 23832123

[31] Y. B. Luan , L. Q. Jing , M. Z. Xie , et al., “Synthesis of Efficient N‐containing TiO2 Photocatalysts With High Anatase Thermal Stability and the Effects of the Nitrogen Residue on the Photoinduced Charge Separation,” Physical Chemistry Chemical Physics 14, no. 4 (2012): 1352–1359, 10.1039/C1CP22907A. 22159028

[32] K. F. Zhang , J. Xu , T. R. Yan , et al., “Molecular Modulation of Sequestered Copper Sites for Efficient Electroreduction of Carbon Dioxide to Methane,” Advanced Functional Materials 33, no. 17 (2023): 2214062, 10.1002/adfm.202214062.

[33] Q. W. Chang , Y. M. Liu , J. H. Lee , et al., “Metal‐Coordinated Phthalocyanines as Platform Molecules for Understanding Isolated Metal Sites in the Electrochemical Reduction of CO2,” Journal of the American Chemical Society 144, no. 35 (2022): 16131–16138, 10.1021/jacs.2c06953. 36007154

[34] D. Yao , C. Tang , X. Zhi , et al., “Inter‐Metal Interaction With a Threshold Effect in NiCu Dual‐Atom Catalysts for CO2 Electroreduction,” Advanced Materials 35, no. 11 (2023): 2209386, 10.1002/adma.202209386. 36433641

[35] Y.‐Y. Lou , Q. I. Z. Zheng , S.‐Y. Zhou , et al., “Phase‐dependent Electrocatalytic Nitrate Reduction to Ammonia on Janus Cu@Ni Tandem Catalyst,” ACS Catalysis 14, no. 7 (2024): 5098–5108, 10.1021/acscatal.4c00479.

[36] Y. Mou , X. D. Wu , C. C. Qin , et al., “Linkage Microenvironment of Azoles‐Related Covalent Organic Frameworks Precisely Regulates Photocatalytic Generation of Hydrogen Peroxide,” Angewandte Chemie International Edition 62, no. 36 (2023): 202309480, 10.1002/anie.202309480. 37462327

[37] Y. Wang , H. C. He , Y. N. Li , et al., “Unraveling the Photo‐Induced Dynamic Behavior of COF‐Based Z‐scheme Heterostructure Monolithic Aerogels,” Matter 7, no. 9 (2024): 3145–3162, 10.1016/j.matt.2024.05.003.

[38] B. Pattengale and J. Huang , “Implicating the Contributions of Surface and Bulk States on Carrier Trapping and Photocurrent Performance of BiVO4 Photoanodes,” Physical Chemistry Chemical Physics 19, no. 9 (2017): 6831–6837, 10.1039/C6CP08564D. 28218314

[39] G. X. Dong , M. R. Zhang , S. X. Yuan , M. Zhang , and T. B. Lu , “Chlorine Radical‐Mediated Photocatalytic C─C Coupling of Methanol to Ethylene Glycol With near‐Unity Selectivity,” Angewandte Chemie International Edition 64, no. 33 (2025): 202510993, 10.1002/anie.202510993. 40501165

[40] C. Xu , S. Yu , M. Y. Zhang , et al., “Synergistic Engineering of Electron‐Enriched Nickel Sites for Highly Efficient Photocatalytic CO2 Reduction to C2H6,” Advanced Functional Materials 35, no. 6 (2025): 2414893, 10.1002/adfm.202414893.

[41] L. Luo , X. Y. Han , K. R. Wang , et al., “Nearly 100% Selective and Visible‐Light‐driven Methane Conversion to Formaldehyde via. Single‐atom Cu and Wδ+,” Nature Communications 14, no. 1 (2023): 2690, 10.1038/s41467-023-38334-7. PMC10172301 37165020

[42] J. Li , H. L. Huang , W. J. Xue , et al., “Self‐adaptive Dual‐metal‐site Pairs in Metal‐organic Frameworks for Selective CO2 Photoreduction to CH4,” Nature Catalysis 4, no. 8 (2021): 719–729, 10.1038/s41929-021-00665-3.

[43] H. Cao , X. Zhu , J. Xue , et al., “Defect‐Mediated Cu–S Pair Active Sites Modulating Proton Supply to Facilitate Overall CO2 Photoreduction With H2O,” ACS Catalysis 14, no. 13 (2024): 9734–9741, 10.1021/acscatal.4c02857.

[44] W. F. Zhang , Y. B. Qi , Y. Zhao , et al., “Rh‐Dispersed Cu Nanowire Catalyst for Boosting Electrocatalytic Hydrogenation of 5‐hydroxymethylfurfural,” Science Bulletin 68, no. 19 (2023): 2190–2199, 10.1016/j.scib.2023.07.036. 37580202

[45] Y. F. Li , C. C. Wang , L. K. Yang , et al., “Enhancement of Nitrate‐to‐Ammonia on Amorphous CeOx‐Modified Cu via Tuning of Active Hydrogen Supply,” Advanced Energy Materials 14, no. 7 (2024): 2303863, 10.1002/aenm.202303863.

[46] X. Sun , Y. He , M. Wang , et al., “Maximizing Available Active Hydrogen on FeNi Substitutional Solid‐Solution Alloy to Boost Electrosynthesis of Ammonia From Nitrate,” ACS Nano 19, no. 8 (2025): 8189–8199, 10.1021/acsnano.4c17163. 39976360

[47] R. J. Zeng , T. Y. Liu , M. H. Qiu , et al., “High‐Volumetric Density Atomic Cobalt on Multishell ZnxCd1–xS Boosts Photocatalytic CO2 Reduction,” Journal of the American Chemical Society 146, no. 14 (2024): 9721–9727, 10.1021/jacs.3c13827. 38556809

[48] Y. X. Liu , J. J. Chen , W. C. Li , et al., “Aqueous Zn–CO2 Batteries: A Route towards Sustainable Energy Storage,” Ind Chem Mater 2, no. 4 (2024): 514–532, 10.1039/D4IM00014E.

[49] Y. Wang , J. X. Wei , H. L. Tang , et al., “Artificial Photosynthetic System for Diluted CO2 Reduction in Gas‐solid Phase,” Nature Communications 15, no. 1 (2024): 8818, 10.1038/s41467-024-53066-y. PMC11470023 39394216

[50] W. T. Song , K. C. Chong , G. B. Qi , et al., “Unraveling the Transformation From Type‐II to Z‐Scheme in Perovskite‐Based Heterostructures for Enhanced Photocatalytic CO2 Reduction,” Journal of the American Chemical Society 146, no. 5 (2024): 3303–3314, 10.1021/jacs.3c12073. 38271212

[51] Y. N. Gong , S. Q. Zhao , H. J. Wang , et al., “A Planar‐Structured Dinuclear Cobalt(II) Complex With Indirect Synergy for Photocatalytic CO2‐to‐CO Conversion,” Angewandte Chemie International Edition 63, no. 45 (2024): 202411639, 10.1002/anie.202411639. 38976517

