# Supplementary material for: Intramolecular Synergy of CO2 Activation and H Spillover on Heteronuclear Dual‐Metal Phthalocyanine Assemblies for Selective CO2 Photoreduction
Source: Adv Sci (Weinh). 2026 Jan 4;13(14):e21954. doi: 10.1002/advs.202521954 (PMC12970181; doi:10.1002/advs.202521954)
Supplement: Supplementary file 1 — Supporting Information: advs73582‐sup‐0001‐SuppMat.pdf [file ADVS-13-e21954-s001.pdf]

Supporting Information

Intramolecular synergy of CO_2 activation and H spillover on heteronuclear dual-metal phthalocyanine assemblies for selective CO_2 photoreduction

Ye Liu, Wei Qin, Panzhe Qiao, Ziqing Zhang, Zhuo Li, Yangyang Zhu, Jianhui Sun, Zhijun Li Fuquan Bai, Liqiang Jing,* and Ji Bian**

1. Chemicals.

1,2-dicyanobenzene [$C_8H_4N_2$, Aladdin, CAS#:91-15-6], 1,2,4,5-tetracyanobenzene [$C_{10}H_2N_4$, Aladdin, CAS#:712-74-3], 1,8-diazabicyclo[5.4.0]undec-7-ene (DBU) [$C_9H_{16}N_2$, Aladdin, CAS#:6674-22-2], Copper(II) chloride [$CuCl_2$, Aladdin, CAS#:7447-39-4], Nickel(II) chloride [$NiCl_2$, RHAWN, CAS#:7718-54-9], Iron(III) chloride [$FeCl_3$, Aladdin, CAS#:7705-08-0], Zinc(II) chloride [$ZnCl_2$, Aladdin, CAS#:7646-85-7], Cobalt (II) chloride [$CoCl_2$, RHAWN, CAS#:7646-79-9], Manganese(II) chloride [$MnCl_2$, Aladdin, CAS#:7773-01-5], 1-Pentanol [$C_5H_{12}O$, Aladdin, CAS#:71-41-0], Bismuth Chloride [$BiCl_3$, Aladdin, CAS#:7787-60-2], Sodium metavanadate [$NaVO_3$, Aladdin, CAS#:19718-26-8], Hexadecyl trimethyl ammonium bromide [$C_{19}H_{42}BrN$, Aladdin, CAS#:57-09-0], Nafion perfluorinated resin [$Rf[OCF_2CF(CF_3)_2]nOCF_2CF_2SO_3H$, RHAWN, CAS#:31175-20-9], Sodium sulfate anhydrous [$NaSO_4$, Aladdin, CAS#: 15124-09-1], Hydrochloric acid [HCl, Kermel, CAS#: 7647-01-0] were used as received without further purification. Methanol and ethanol were purchased from Tianjin Fuyu Fine Chemical Co. Ltd. High purity carbon dioxide (CO_2) and nitrogen (N_2) gas were bought from Harbin Qing Hua Industrial Gas Co. Ltd. Ultrapure deionized water (18.2 MΩ/cm) was used in all experiments.

2. Experimental Section

Synthesis of CuNiPc: The synthesis procedure of CuNiPc was adopted from the previous publication.[1] Specially, 1.7 mmol of 1,2-dicyanobenzene, 0.3 mmol of 1,2,4,5-tetracyanobenzene, 0.54 mmol of $CuCl_2$ and 0.36 mmol of $NiCl_2$ were transferred to a 100 mL three-necked flask, followed by addition of 20 mL of n-pentanol as the reaction solvent. After several cycles of vacuum and Ar gas, 0.3 mL of DBU was added to the above solution. The mixture was then heated to 140°C under Ar flow for 12 h to obtain a dark blue powder. After cooling to room temperature, 200 mL of ethanol and methanol were added to dissolve the unreacted reactants under stirring at 500 rpm for 30 min at 70°C, respectively. Subsequently, the precipitate was collected and stirred in HCl (1 mol/L) for 12 h at room temperature. The crude product was further filtered, washed with water, ethanol and then purified by Soxhlet extraction with acetone. Finally, the precipitate was lyophilized to yield the final product. The samples with different Cu/Ni ratios were obtained by altering the used dosage of Cu and Ni sources, and denoted as Cu_xNi_yPc, where the x/y indicates the molar ratio of $CuCl_2/NiCl_2$. The $Cu_{1.5}Ni_1Pc$ one is denoted as CuNiPc for short. Other heteronuclear metal phthalocyanines (CuFePc, CuMnPc, CuCoPc and CuZnPc) were synthesized by the identical procedure but the $NiCl_2$ was replaced by $FeCl_3$, $MnCl_2$, $CoCl_2$ and $ZnCl_2$, respectively.

Synthesis of CuCuPc: CuCuPc and NiNiPc were synthesized by the identical method as CuNiPc. Specifically, 1.7 mmol of 1,2-dicyanobenzene, 0.3 mmol of 1,2,4,5-tetracyanobenzene, 0.9 mmol of $CuCl_2$ were transferred to a 100 mL three-necked flask, followed by addition of 20 mL of n-pentanol as the reaction solvent. After several cycles of vacuum and Ar gas, 0.3 mL of DBU was added to the above solution. The mixture was then heated to 140°C under Ar flow for 12 h to obtain a dark blue powder and followed by the purification. NiNiPc was synthesized using the same procedures but the $CuCl_2$ was replaced by $NiCl_2$.

Synthesis of CuPc: CuPc was synthesized using a previously reported method[2] with a slight modification. Specifically, 4 mmol of 1,2-dicyanobenzene and 1 mmol of $CuCl_2$ were dispersed and dissolved in 20 mL of n-pentanol in a 100 mL round-bottom flask. After several cycles of vacuum and Ar gas, 0.3 mL of DBU was added to the above solution. The mixture was then heated to140°C under Ar flow for 12 h to obtain a dark blue powder, and followed by purification. NiPc was synthesized using the same procedures but the $CuCl_2$ was replaced by $NiCl_2$.

Synthesis of $BiVO_4$ nanosheets: In a typical synthesis, 2.21 g of $BiCl_3$ and 1.05 g of cetyltrimethylammonium bromide (CTAB) were dissolved in 60 mL of ethylene glycol. Then, 0.86 g $NaVO_3$ was added to the above solution, stirred for 30 min and transferred into a 100 mL polytetrafluoroethylene reactor and heated at 120°C for 12 h. After that, the reactor was cooled to room temperature, and the resulting products were washed with ethanol and ultrapure deionized water several times until a yellow solid was obtained, and then dried at 60°C in a vacuum oven. Finally, the obtained sample was calcined for 8 min at 450°C and denoted as $BiVO_4$.

Synthesis of CuNiPc/$BiVO_4$ heterojunctions: A certain amount of CuNiPc and $BiVO_4$ was dispersed in 70 mL of ethanol, ultrasonicated for 30 min and stirred at room temperature overnight. After that, the mixture was heated at 80°C for 6 h in a water bath. After the solvent had evaporated, the products were dried under vacuum at 80°C. The obtained samples were denoted as xCuNiPc/$BiVO_4$, where x (0.5, 1.0 and 1.5) represents the mass ratio percentage of CuNiPc to $BiVO_4$. In particular, 1.0CuNiPc/$BiVO_4$ is denoted as CuNiPc/$BiVO_4$ for short.

Characterizations

The X-ray powder diffraction (XRD) patterns of the samples were obtained using a Bruker D8 Advance diffractometer with Cu Kα radiation. Ultraviolet-visible absorption spectra (DRS) were acquired on a Model Shimadzu UV 2700 spectrophotometer, using $BaSO_4$ as the reference. Fourier-transform infrared (FT-IR) spectra were recorded using a Thermo Scientific Nicolet iS50, with KBr as the diluent. Raman spectra were carried out on a HR-800 (Horiba Jobin Yvon LabRam) using a laser of 633 nm and 532 nm. Inductively coupled plasma optical emission spectrometer (ICP-OES) was used to measure element content on Aglient 5110. The thickness of the samples was tested by atomic force microscopy (AFM) using a multi-mode nanoscope VIII instrument (Bruker) and a silicon wafer (1 cm × 1 cm) as the base. Transmission electron microscopy (TEM) images were obtained using a JEOL JEM-F200 instrument with an acceleration voltage of 200 kV. The elemental composition of the samples was evaluated using an Energy Dispersive Spectrometer (EDS). X-ray photoelectron spectroscopy (XPS) was performed using the Thermo Scientific ESCALAB 250Xi instrument with an Al Kα radiation source. In-situ XPS spectra were measured on the Thermo Scientific ESCALAB 250Xi instrument with an Al Kα radiation source, where the data was collected before light irradiation or after being irradiated for 10 min. The X-ray absorption data at the Cu and Ni K-edge of the samples were measured at room temperature in transmission mode using ion chambers (and in the fluorescent mode with the 4-element SDD) at the Beamline BL20U1 of Shanghai Synchrotron Radiation Facility (SSRF, China). The station was operated with a Si (111) double crystal monochromator. During the measurement, the storage ring was operated at the energy of 3.5 GeV and a current of 200 mA (top-up). The photon energy was calibrated with the first inflection point in Cu and Ni K-edge metal foil. Photoluminescence spectroscopy was performed on fluorescence spectrophotometer (Perkinelmer, LS 55). Steady-state surface photovoltage spectroscopy (SS-SPS) measurements were conducted on a home-built apparatus equipped with a lock-in amplifier (SR830, USA) synchronized with a light chopper (SR540, USA). Electron paramagnetic resonance (EPR) measurements were conducted using a Bruker EMX plus model spectrometer.

Photoelectrochemical and electrochemical measurements: The film electrode was fabricated as follows: 10 mg of samples, 20 μL of Nafion and 180 μL of ethanol were mixed thoroughly into the slurry. The slurry was then coated on the FTO glass electrode (1.0 cm × 1.0 cm). Finally, the coated electrode was dried at 80°C for 2 h. Photoelectrochemical (PEC) and electrochemical (EC) measurements were conducted on an IVIUM V13806 electrochemical workstation with a

three-electrode system. The as-prepared film electrodes were used as working electrodes, and a Pt plate (99.9%) and Ag/AgCl were used as the counter and reference electrodes, respectively. 0.2 mol/L Na_2SO_4 solution was used as the electrolyte for all PEC measurements. Mott-Schottky plots were implemented at frequencies of 500, 800, and 1100 Hz. I-V curves were obtained under irradiation by a 300 W Xenon lamp. EC experiments were performed in a high-purity N_2 or CO_2 bubbled system.

Hydroxyl radical measurement: 0.02 g of samples was dispersed in a 100 mL beaker containing 50 mL coumarin aqueous solution (1 mM). Prior to irradiation, the solution was stirred magnetically for 30 min to attain an adsorption-desorption equilibrium in dark. The solution was then irradiated for 1 h using a 150 W Xenon lamp (GYZ220, China). After irradiation, the sample was centrifuged, and then a certain volume of solution was transferred into a Pyrex glass cell for the fluorescence measurement. The fluorescence of 7-hydroxycoumarin was detected using a spectrofluorometer at an excitation wavelength of 350 nm, with the emission peak observed at 460 nm.

In-situ EPR measurements: CuNiPc/$BiVO_4$ was placed in a paramagnetic tube and then immobilized on EPR resonator. The sample was vacuumed to remove the air in the tube. After that, the paramagnetic tube was sealed with a rubber stopper and sealing film. The data were recorded before and after irradiation with a 300 W Xe lamp, respectively. After that, the CO_2 was introduced for approximately twenty minutes, and the EPR signals under illumination were collected again.

In-situ XAFS measurement: The photochemical in situ XAFS tests were performed by a professional photocatalytic cell. This in-situ cell uses quartz glass as the xenon lamp light inlet window to ensure the effective irradiation of the sample by simulating the full band of sunlight, and uses beryllium as the X-ray window material to ensure the minimum attenuation of X-rays and the overall airtightness of the cell. The XAFS spectra were collected through fluorescence mode with the 4-element SDD. The CuNiPc/$BiVO_4$ catalyst was pressed into thin sheets by calculating the optimal mass and then placed in the sample slot of the cell. To obtain the key information on the evolution of active sites during photochemical reactions, a series of representative working conditions were applied to the samples, including Ar-saturated solution (In Ar), CO_2 saturated solution (In CO_2) and light conditions (300W Xe lamp, CEL-HXF300-S, CEAulight) with CO_2 saturated solution.

In-situ diffuse reflectance infrared Fourier transform (DRIFTS): To investigate reactant adsorption and reaction intermediates during the CO_2 reduction process, in-situ DRIFTS was conducted under mimicking conditions with the reaction. The sample was firstly loaded in the sample holder with a flat surface. A cover was then secured on the sample holder to form a reaction cell connected to an evacuation line. The reaction cell was evacuated to remove all adsorbed impurities and subsequently purged with 25% CO_2 gas flow (diluted by N_2) carrying by water. After the equilibrium of CO_2 and water adsorption on the photocatalyst was reached, the data was collected as the background. Subsequently, UV-vis light was introduced to the reaction space via an observation window and the in-situ DRIFTS data was collected at specified intervals.

Femtosecond-transient absorption spectroscopy (fs-TAS): The femtosecond TAS measurements were performed using a regenerative amplified Ti: sapphire laser system (Coherent; 800 nm, 70 fs, 6 mJ /pulse, and 1 kHz repetition rate) as the laser source and a femto-TA100 spectrometer (Time-Tech Spectra). Briefly, the 800 nm output pulse from the regenerative amplifier was split in two parts with a 50% beam splitter. The transmitted part was used to pump an Optical Parametric Amplifier (OPA) which generated a wavelength of 355 nm as pump beam. The reflected 800 nm beam was split again into two parts. One part with less than 10% was attenuated with a neutral density filter and focused into a 2 mm thick sapphire window to generate a white light continuum (WLC) used for probe beam. The probe beam was focused with an Al parabolic reflector onto the sample. After the sample, the probe beam was collimated and then focused into a fiber-coupled spectrometer with CMOS sensors and detected at a frequency of 1 KHz. The delay between the pump and probe pulses was controlled by a motorized delay stage. The pump pulses were chopped by a synchronized chopper at 500 Hz and the absorbance change was calculated with two adjacent probe pulses (pump-blocked and pump-unblocked).

Photocatalytic CO_2 reduction performance test: The photocatalytic reaction was conducted on a 100 mL quartz cell reactor equipped with a 300 W xenon lamp as the light source. The as-prepared catalyst (10 mg) was suspended in 10 mL of water under magnetic stirring. High-purity CO_2 gas (99.9%) was passed through water and then into the reaction setup to reach ambient pressure. The photocatalyst was allowed to equilibrate in the CO_2/H_2O system for 30 min and then under light irradiation. The gaseous products were analyzed online every hour using a gas chromatograph equipped with both TCD and FID detectors (GC-7920, Au Light).

The isotope labelling experiment was carried out using $^{13}CO_2$ instead of CO_2, and the reduction products were analyzed by gas chromatography-mass spectrometry (GC-MS, MS5977A, Agilent). The stability and recyclability tests were performed for 4 cycles, with each cycle lasting 4 h. After one cycle, the samples were recovered by centrifugation, washed, and dried before being used in the next cycle.

The selectivity of CO production was calculated using the following equation (1):

$$\text{CO selectivity} = \frac{\text{R(CO)}\times 2}{\text{R(CO)}\times 2+\text{R(CH}_4)\times 8} \quad (1)$$

where R(CO) and R(CH_4) represent the evolution rate of CO and CH_4, respectively.

3. Computational models and methods.

In this study, all calculations for the relevant properties of phthalocyanine molecules were performed using Gaussian16 program package. Systematic structural optimizations of phthalocyanine molecules were conducted based on density functional theory (DFT). Following literature analysis and comprehensive functional tests, and considering the characteristics of phthalocyanine systems, we selected the PBE0 hybrid functional for calculation. The functional has been extensively validated in numbers studies to accurately describe the electronic structure, absorption spectra and other properties of phthalocyanine molecules.[3-5] Regarding basis set selection, for non-metallic atoms within the phthalocyanine molecules studied, the 6-31G (d,p) description was used, while the LANL2DZ pseudopotential basis set was used for metal atoms.[6-8] This choice of method ensures a balance between the accuracy of the electronic structure description and computational efficiency throughout this work.

To investigate the interaction mechanisms between phthalocyanine molecules, this study further calculated the UV-Vis absorption spectra of phthalocyanine dimers.[9-11] The same theoretical method (PBE0/6-31G(d,p)/LANL2DZ) as used for monomers was applied in the calculations. By comparing the spectral characteristics between dimers and monomers, we conducted an in-depth analysis of the stacking patterns and intermolecular interaction nature of phthalocyanine molecules.

For the investigation of the catalytic performance in phthalocyanine/$BiVO_4$ heterojunction structure, we conducted relevant calculations using Gaussian16 package. Since the CO_2 reduction reaction (CO_2RR) mainly occurs on the phthalocyanine molecules, and considering computational resources limitations and efficiency, we simplified the model system. This model infers the catalytic performance of the heterojunction for CO_2RR by comparing the reaction energy barrier for CO_2 on the isolated phthalocyanine molecule. Within these calculations, the effect of simulated light conditions and solvation (modeled using the SMD implicit solvent

model for an aqueous environment) were incorporated. Based on experimental findings and literature studies, the CO_2RR process was carried out in three steps, with the total and stepwise reactions as follows [12, 13]:

$$CO_2 + H^+ + e^- \rightarrow CO + H_2O \qquad \text{(step 1)}$$

$$* + CO_2 + H^+ + e^- \rightarrow * COOH \qquad \text{(step 2)}$$

$$* COOH + H^+ + e^- \rightarrow * CO + H_2O \qquad \text{(step 3)}$$

$$* CO = * + CO \qquad \text{(step 4)}$$

The adsorption energy equation can be expressed as:

$$E_{ads} = E_{(*+mole)} + E_* + E_{mole} \qquad (2)$$

Where E* denotes the energy of the catalyst phthalocyanine molecule, E(*+mole) denotes the total energy of the adsorbed intermediates on the catalyst surface, and Emole denotes the energy of the small molecule.

The time-dependent survival probabilities (TDSP) curves were defined as the probability of the photo-excited electron which is still in the adsorbed dye molecule at time t.[14-17] Therefore, the TDSP can be computed by applying the time-evolved electronic wave function into the atomic orbitals of the adsorbed dye molecule. The detailed information of the ultrafast interfacial electron transfer was provided according to the semi-empirical extended Hückel (EH) theory to perform semi-classical quantum dynamics simulations. The most important improvement of EH is that the Hamiltonian includes not only the π orbital but also the σ one formed by s, p and d atomic orbitals to represent a full valence shell.

Supporting Figures

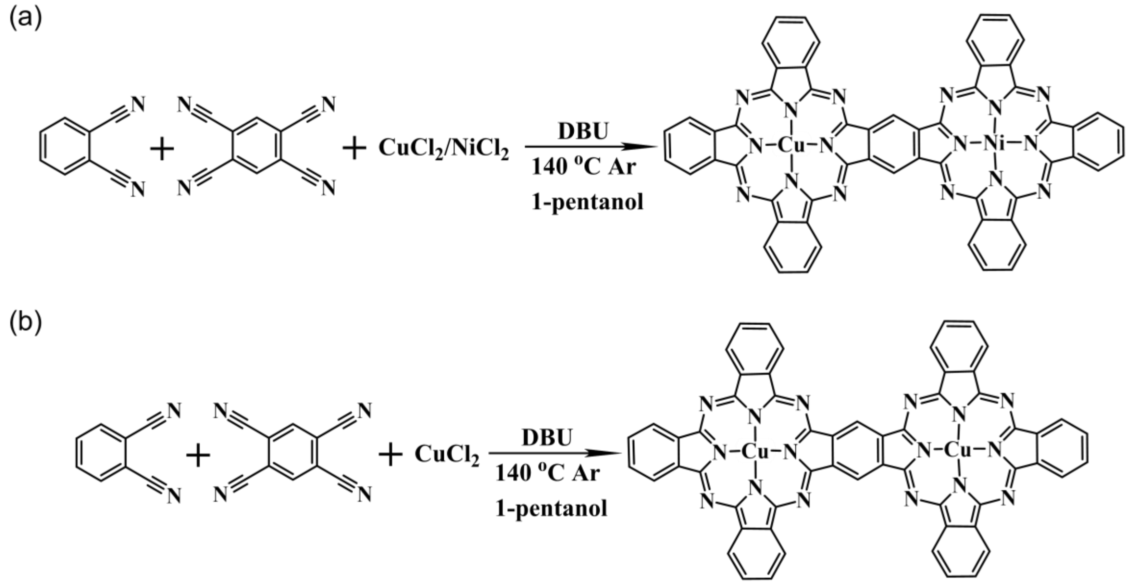

Figure S1 Synthesis routes of CuNiPc (a) and CuCuPc (b).

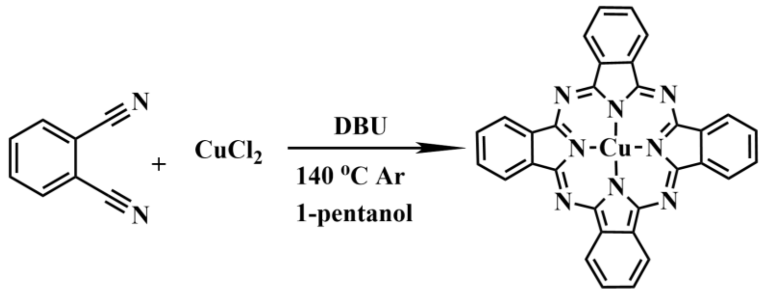

Figure S2 Synthesis route of CuPc.

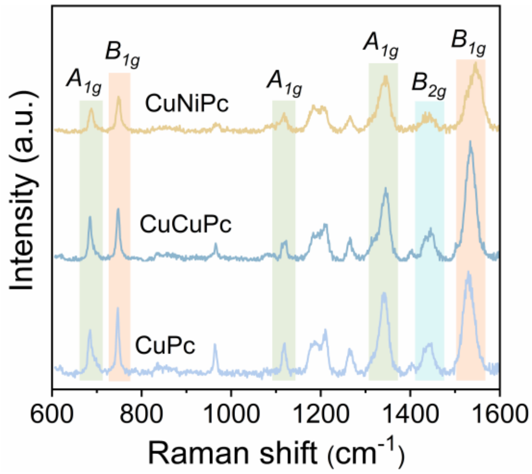

Figure S3 Raman spectra of CuNiPc, CuCuPc and CuPc.

Note: The Raman spectra of CuNiPc, CuCuPc and CuPc exhibits characteristic fingerprint bands at 686, 747, 1118, 1344, 1444 and 1534 cm^{-1}, respectively.[18] Notably, the A_{1g} mode (at 1344 cm^{-1}) and B_{1g} mode (at 1444 cm^{-1}) of CuNiPc and CuCuPc showed an obvious shift to high-frequency in comparison with CuPc (at 1341 and 1441 cm^{-1}), which can be attributed to strong planar electron delocalization in the phthalocyanine macrocycle.

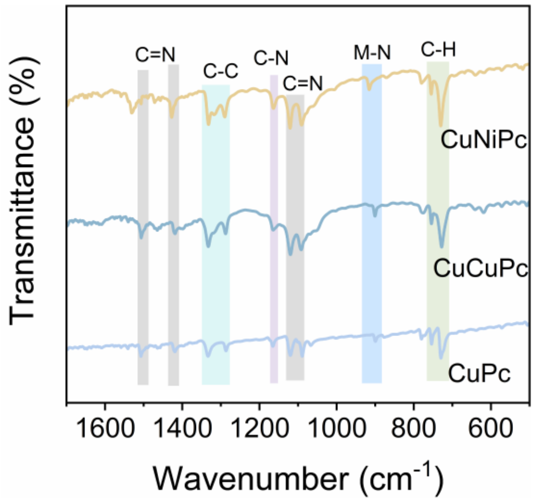

Figure S4 FT-IR spectra of CuNiPc, CuCuPc and CuPc.

Note: The typical peaks corresponding to the phthalocyanine skeleton vibration (1087,1332 and 1530 cm^{-1}),[18] stretching vibration of C–H (728 and 755 cm^{-1})[19] and metal–ligand bond (M–N) vibration (901 cm^{-1})[20] are observed in the Fourier transform infrared (FT-IR) spectra of CuNiPc, CuCuPc and CuPc.

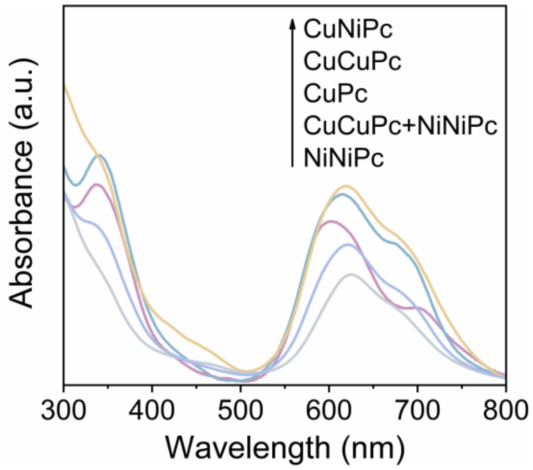

Figure S5 UV-vis absorption spectra of CuNiPc, CuCuPc, CuPc, CuCuPc+NiNiPc and NiNiPc.

Note: UV-visible (UV-vis) absorption spectra explored the intrinsic light-harvesting properties of CuNiPc, CuCuPc, CuPc, CuCuPc+NiNiPc mixture and NiNiPc monomers in N, N-dimethylformamide (DMF). The absorption spectrum of phthalocyanines exhibit two typical characteristic bands: the Q band in the range of 600-750 nm, attributed to the $a_{1u}(\pi)\rightarrow e_g(\pi^*)$ transition from the highest occupied molecular orbital to the lowest unoccupied molecular orbital (LUMO), and the Soret band (B band) in the range of 300-400 nm, corresponding to the deeper $a_{2u}(\pi)\rightarrow$LUMO transition.[21] Notably, the Q band absorption exhibits a redshift in CuCuPc relative to CuPc, which is attributed to the extended conjugate structure.[21] In addition, for binuclear phthalocyanines, the Q band absorption peaks for CuCuPc (615 nm) and NiNiPc (626 nm) are distinct from CuNiPc (620 nm). The absorption intensities of the Q band for CuNiPc, CuCuPc, CuPc and NiNiPc are different at same concentration. CuNiPc exhibits the strongest light absorption capability among all the phthalocyanines, especially distinct from the mixture of CuCuPc and NiNiPc.

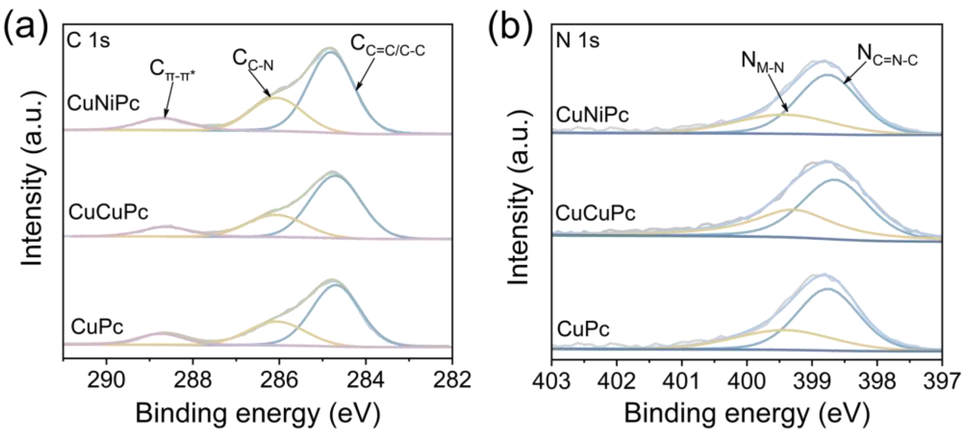

Figure S6 XPS analyses of C 1s (a) and N 1s (b) over CuNiPc, CuCuPc and CuPc.

Note: The chemical states of phthalocyanines were analyzed by X-ray photoelectron spectroscopy (XPS). The C 1s XPS profile displays three deconvoluted peaks, corresponding to C–C/C=C (284.8 eV), C–N (286.1 eV) and the π-π* satellite (288.7 eV), respectively.[18] For the N 1s XPS profile, two peaks are observed at 399.5 eV and 398.8 eV, which are assigned to Metal–N and C–N=C bonds in the phthalocyanine ring, respectively.[19]

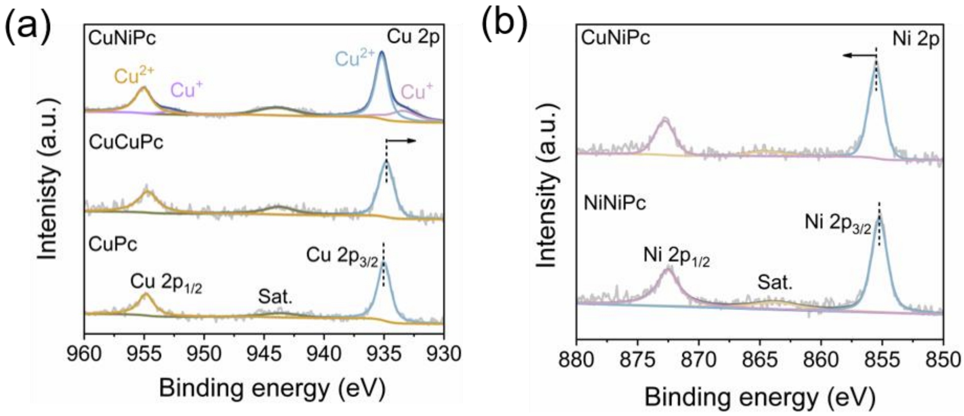

Figure S7 (a) XPS analyses of Cu 2p over CuNiPc, CuCuPc and CuPc. (b) XPS analyses of Ni 2p over CuNiPc and NiNiPc.

Note: The Cu $2p_{3/2}$ and Cu $2p_{1/2}$ high-resolution peaks at approximately 935.04 and 954.85 eV indicate the presence of Cu^{2+} in CuPc.[22] Notably, the binding energy (BE) position of Cu 2p (934.84 and 954.75 eV) in CuCuPc exhibits a slight negative shift compared to that in CuPc, indicating a lower valence state of Cu centers. This difference can be attributed to the extended conjugate structure in binuclear phthalocyanine.[23] Interestingly, the high-resolution peaks corresponding to Cu^{+} (933.10 and 952.61 eV) are also observed on CuNiPc after the incorporation of Ni.[24] In addition, the Ni 2p XPS profile of CuNiPc and NiNiPc indicates that the Ni centers are in the divalent state.[25] Moreover, a positive shift of 0.25 eV for Ni 2p is observed over CuNiPc compared with that of NiNiPc. The result suggests that there is a long-range interaction between the Cu and Ni atoms in CuNiPc, which could alter the electronic state of active sites due to the aromatically conjugated skeleton. This result is consistent with previous studies.[19, 26] Accordingly, it can be confirmed that heteronuclear CuNiPc has been successfully synthesized.

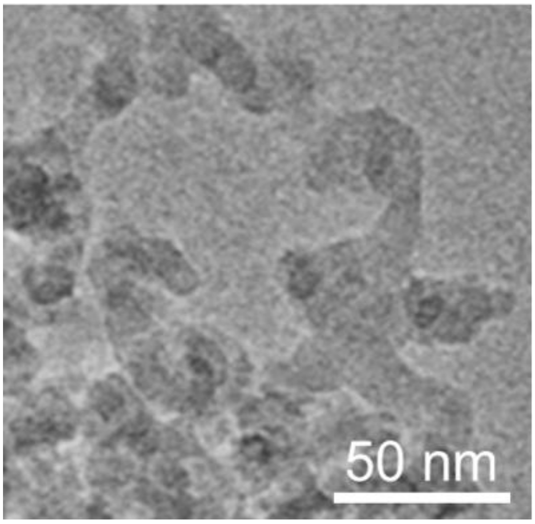

Figure S8 The TEM image of $BiVO_4$.

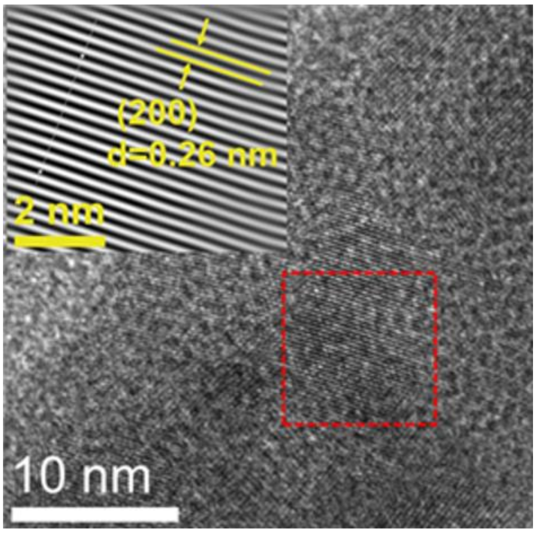

Figure S9 The HRTEM image of CuNiPc/$BiVO_4$.

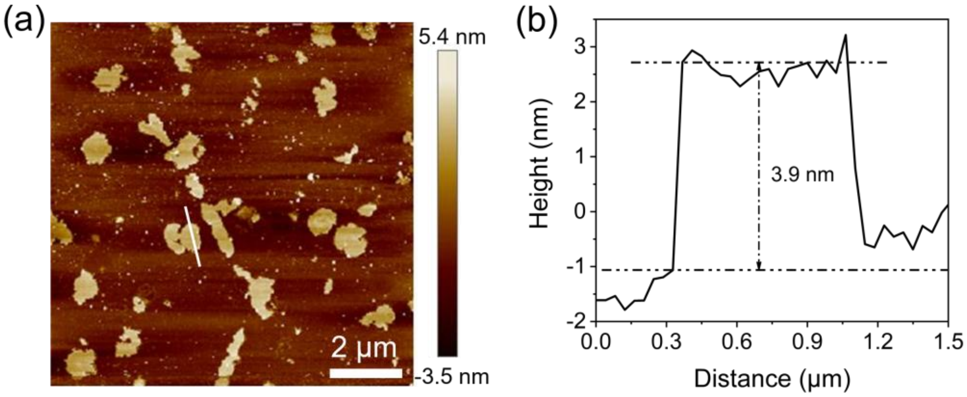

Figure S10 The AFM image (a) and the corresponding height profile (b) of $BiVO_4$.

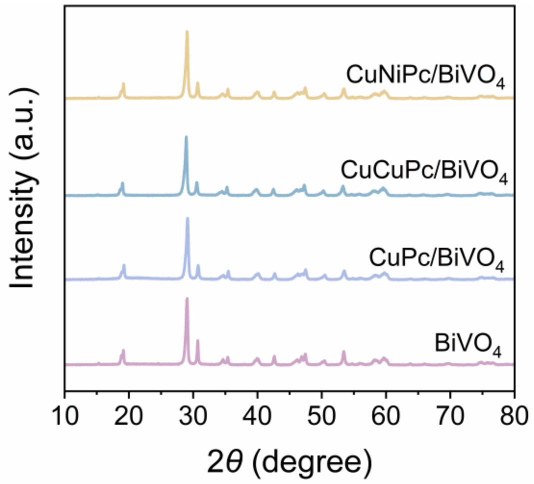

Figure S11 XRD patterns of CuNiPc/$BiVO_4$, CuCuPc/$BiVO_4$, CuPc/$BiVO_4$ and $BiVO_4$.

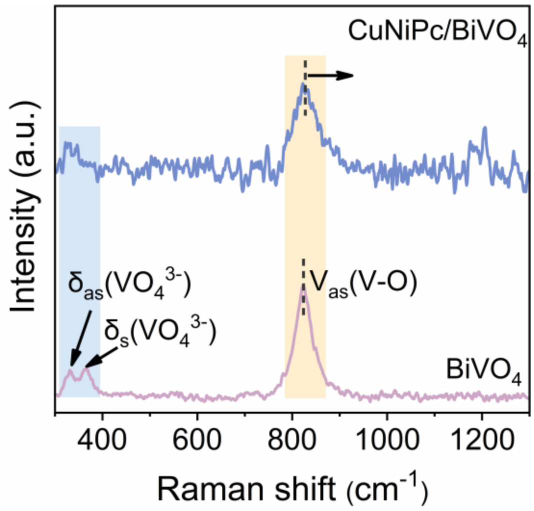

Figure S12 Raman spectra of CuNiPc/$BiVO_4$ and $BiVO_4$.

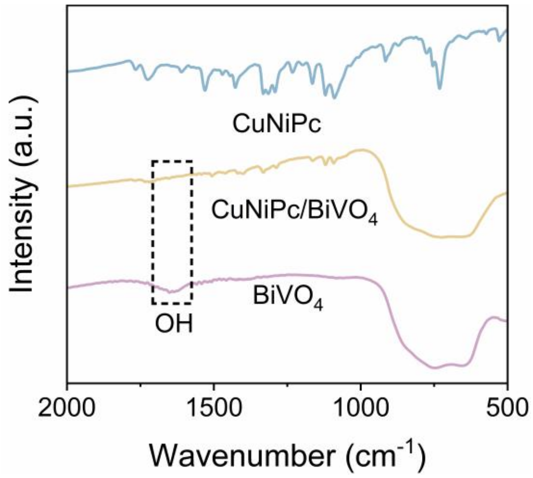

Figure S13 FT-IR spectra of CuNiPc/$BiVO_4$, CuNiPc and $BiVO_4$.

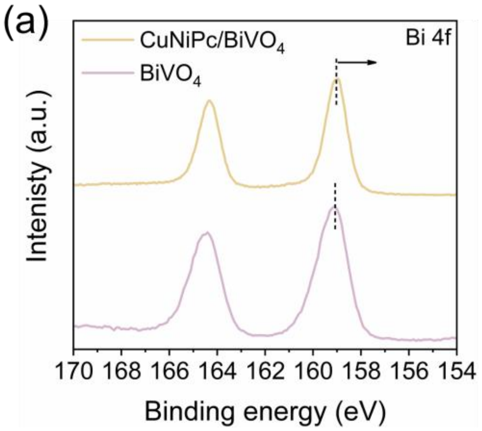

Figure S14 XPS analyses of Bi 4f (a) and V 2p (b) over CuNiPc/$BiVO_4$ and $BiVO_4$.

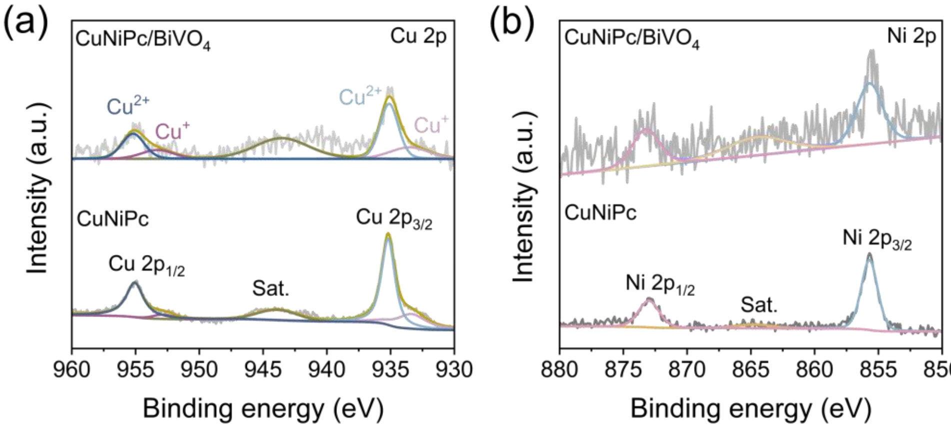

Figure S15 XPS analyses of Cu 2p (a) and Ni 2p (b) over CuNiPc/$BiVO_4$ and CuNiPc.

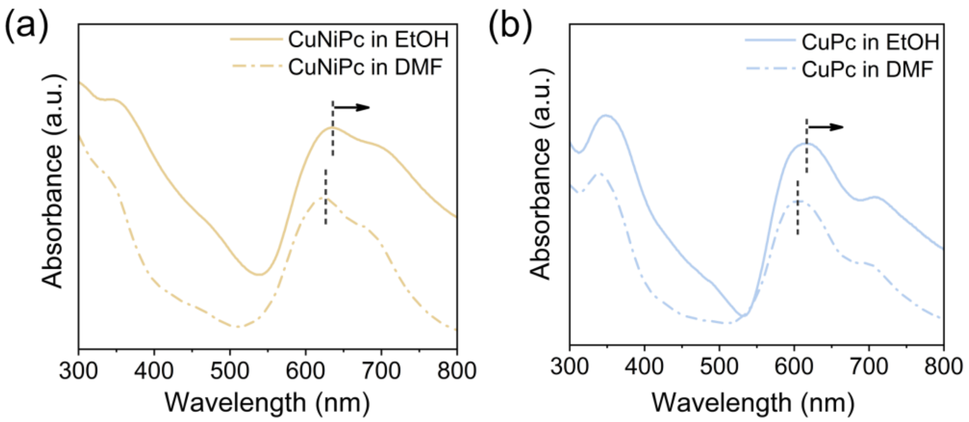

Figure S16 The UV-vis absorption spectra of CuNiPc (a) and CuPc (b) in ethanol (EtOH) and N, N-dimethyl formamide (DMF).

Note: The absorbance spectra reveal that the Q band absorption maxima of CuNiPc and CuPc in ethanol exhibits a red-shift compared to that of phthalocyanine monomers in N, N-dimethylformamide (DMF). The spectroscopic features confirm the predominant formation of J-aggregates for both CuNiPc and CuPc in ethanol.[27]

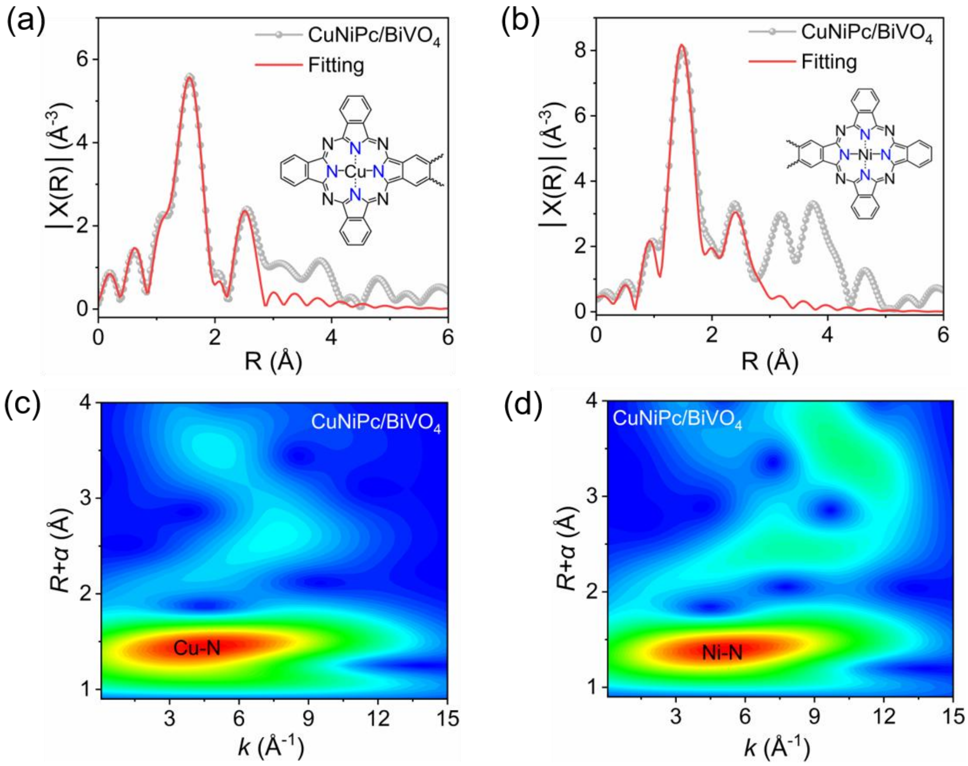

Figure S17 The fitting curve of (a) Cu-N_4 and (b) Ni-N_4 over CuNiPc/$BiVO_4$. (c) Cu and (d) Ni k-edge WT-EXAFS spectra of CuNiPc/$BiVO_4$.

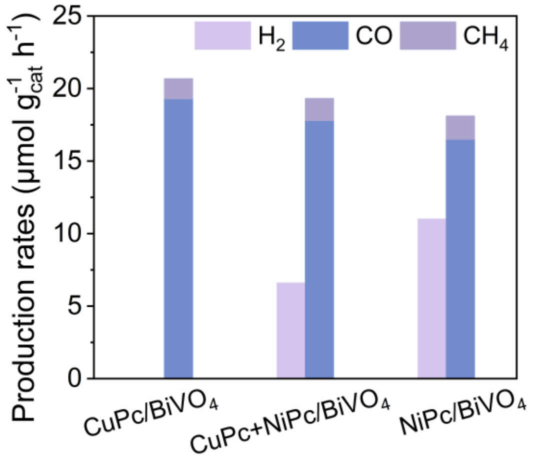

Figure S18 Photocatalytic CO_2 reduction performance of CuPc/$BiVO_4$, CuPc+NiPc/$BiVO_4$ and NiPc/$BiVO_4$ under light irradiation.

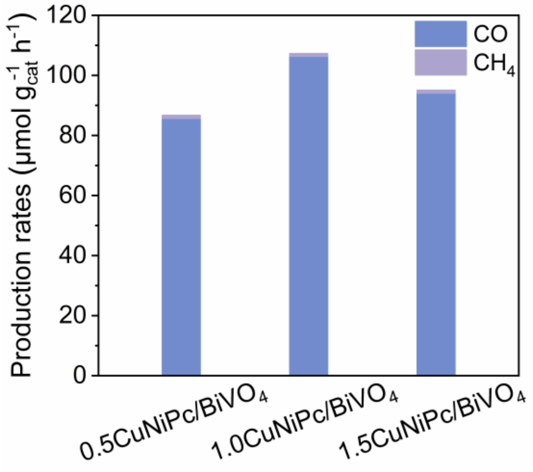

Figure S19 Photocatalytic CO_2 reduction performance of xCuNiPc/$BiVO_4$ under light irradiation (x=0.5, 1.0 and 1.5 represents the mass ratio percentage of CuNiPc to $BiVO_4$).

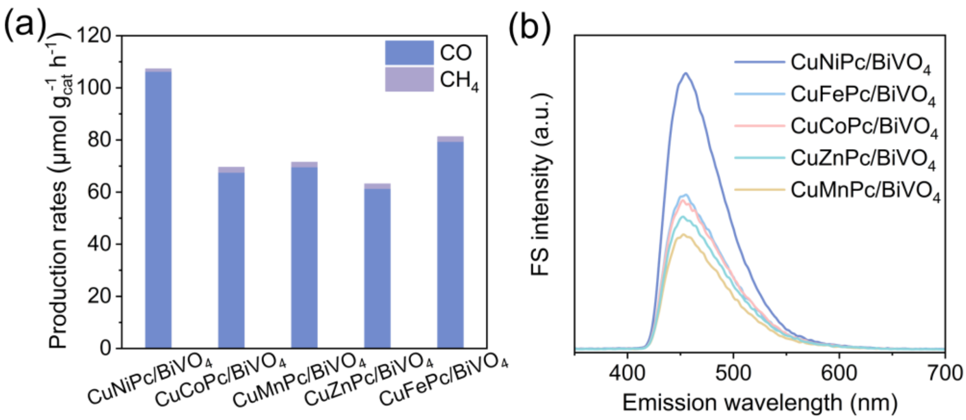

Figure S20 (a) Photocatalytic CO_2 reduction performance and (b) FS spectra related to the formed •OH amounts of CuNiPc/$BiVO_4$, CuCoPc/$BiVO_4$, CuMnPc/$BiVO_4$, CuZnPc/$BiVO_4$ and CuFePc/$BiVO_4$ under light irradiation.

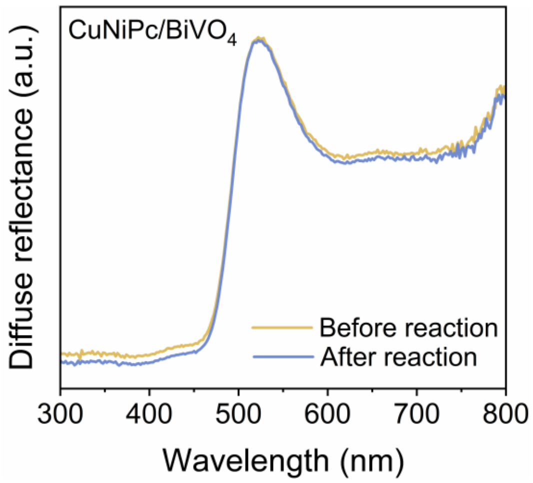

Figure S21 UV-vis diffuse reflection spectra for CuNiPc/$BiVO_4$ before and after photocatalytic CO_2 reduction.

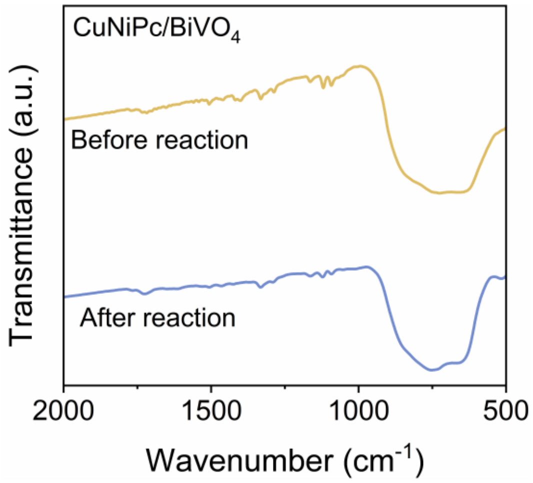

Figure S22 FT-IR spectra for CuNiPc/$BiVO_4$ before and after photocatalytic CO_2 reduction.

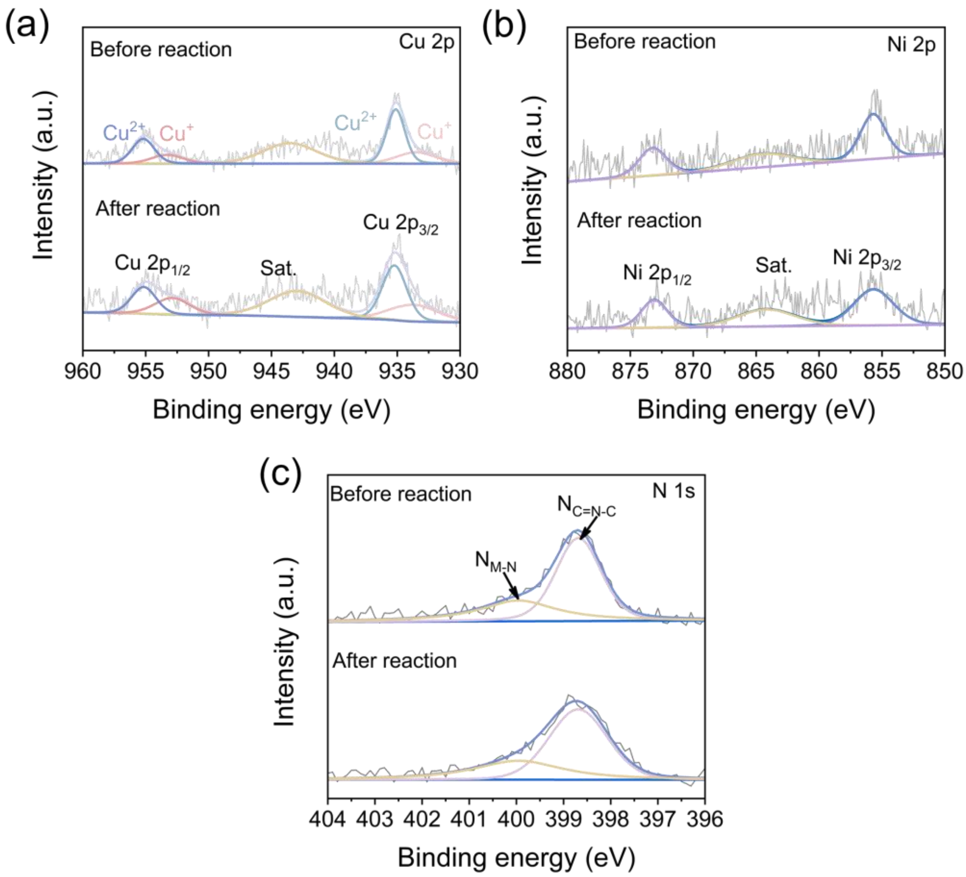

Figure S23 The XPS analyses of (a) Cu 2p, (b) Ni 2p and (c) N 1s for CuNiPc/$BiVO_4$ before and after photocatalytic CO_2 reduction.

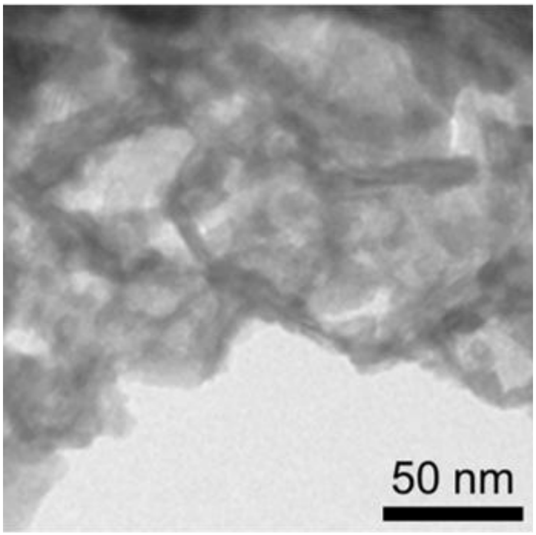

Figure S24 TEM image of CuNiPc/$BiVO_4$ after the reaction.

Note: The DRS spectra, FT-IR spectra and XPS analyses were conducted to investigate the stability of the catalyst. As shown in Figure S21, the optical property of CuNiPc/$BiVO_4$ after the reaction remains unchanged. Besides, the typical peaks corresponding to the phthalocyanine skeleton vibration are observed after the reaction (Figure S22), which indicates the CuNiPc is not decomposed under light irradiation. Moreover, the chemical states of Cu, Ni and N in the CuNiPc/$BiVO_4$ have barely changed after reactions (Figure S23), indicating the structure of CuNiPc has been well retained during the photocatalytic reaction.

In addition, the morphology of CuNiPc/$BiVO_4$ after the reaction (Figure S24) shows that the amorphous CuNiPc is still well dispersed on the $BiVO_4$ nanosheets without aggregation. In combination of DRS spectra, FT-IR spectra, XPS analyses and TEM image, it can be confirmed that the fabricated catalyst is stable during the reaction.

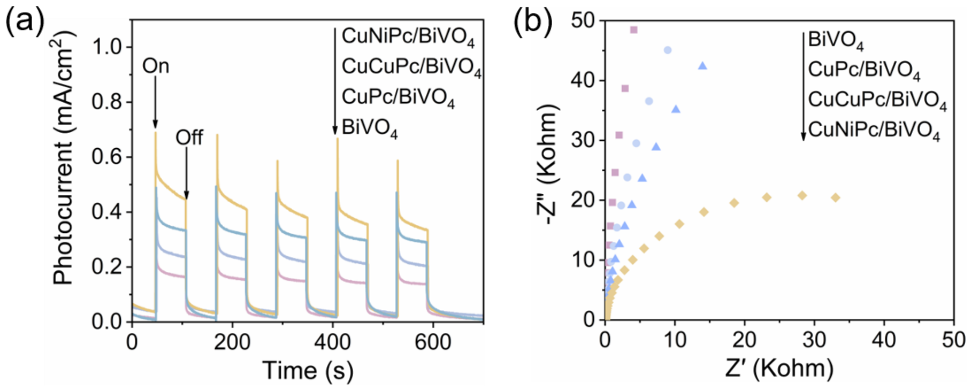

Figure S25 (a) Photocurrent response and (b) electrochemical impedance spectroscopy (EIS) of CuNiPc/$BiVO_4$, CuCuPc/$BiVO_4$, CuPc/$BiVO_4$ and $BiVO_4$.

Note: The photocurrent response and electrochemical impedance spectroscopy (EIS) were employed to investigate the charge transfer and separation of samples. The photocurrent density increases after the introduction of phthalocyanines, in which the CuNiPc/$BiVO_4$ displays the strongest photocurrent response, further confirming the enhancement of photoelectron separation and migration. Similarly, the charge migration improvement is also demonstrated by electrochemical impedance spectroscopy analyses. Smaller impedance radius of heterojunctions is observed in comparison to pristine $BiVO_4$, demonstrating the superior separation and transport in those metal phthalocyanines modified ones. One can see the smallest impedance radius is observed on CuNiPc/$BiVO_4$, implying the best charge separation.

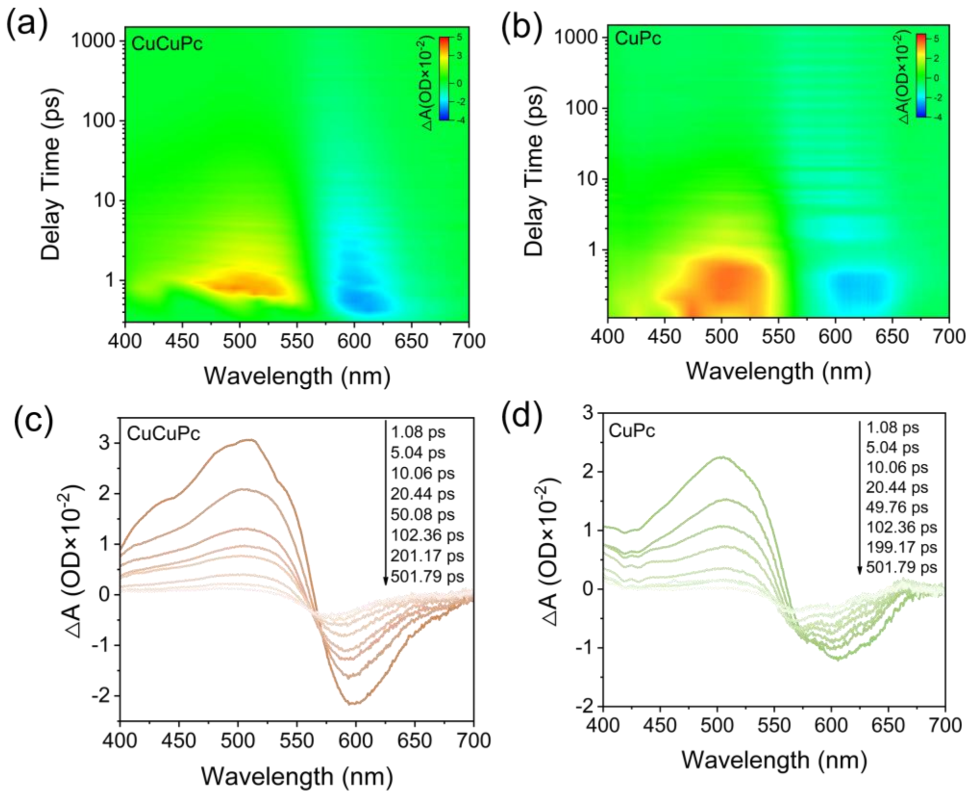

Figure S26 Femtosecond transient absorption (fs-TA) contour maps and spectra at various delay times for CuCuPc (a, c) and CuPc (b, d) following 355 nm laser excitation.

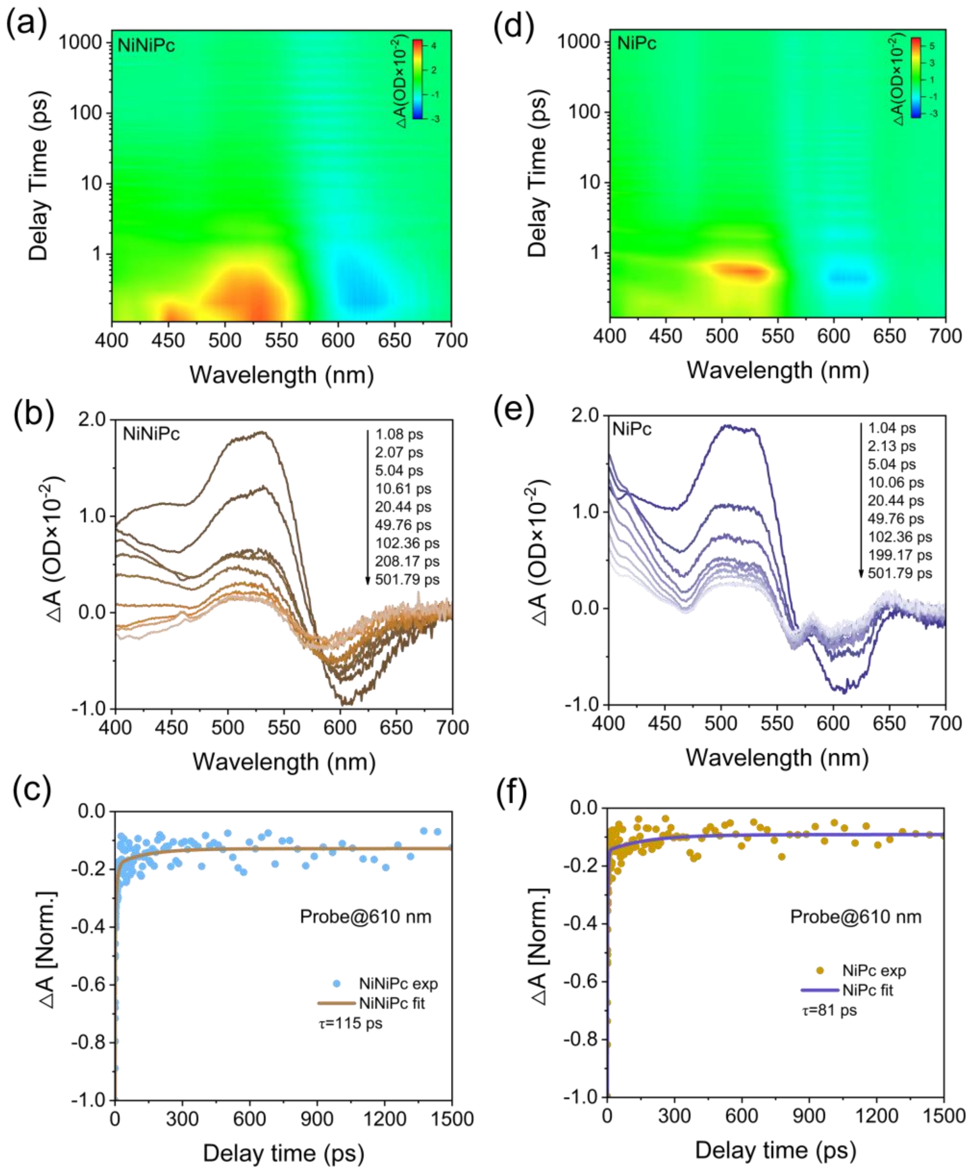

Figure S27 Fs-TA contour maps and spectra at various delay times for NiNiPc (a, b) and NiPc (d, e) following 355 nm laser excitation. The corresponding kinetic decay traces of characteristic signals (c, f) at 610 nm.

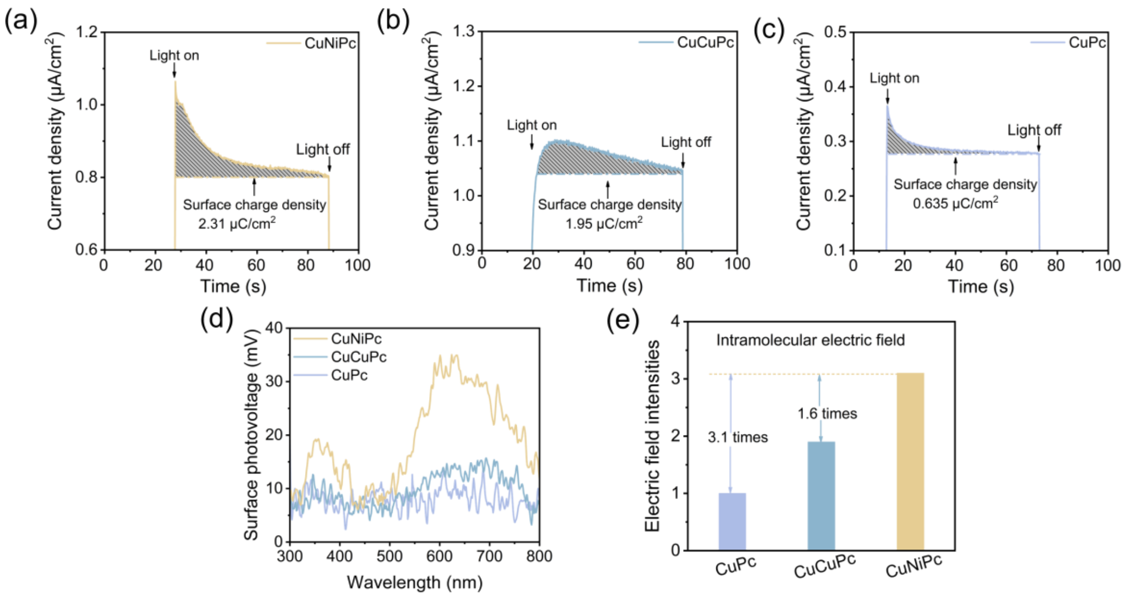

Figure S28 Surface charge densities of (a) CuNiPc, (b) CuCuPc and (c) CuPc. Surface photovoltage (d) and electric field intensities (e) of CuNiPc, CuCuPc and CuPc.

Note: The intramolecular electric field (IEF) of phthalocyanines was investigated by the integrate examination of surface charge density and surface photovoltage. CuNiPc film exhibits a significantly higher surface photovoltage value compared to CuCuPc and CuPc, indicating more efficient charge transport property of CuNiPc. As illustrated in Figure S26, CuNiPc exhibits the strongest IEF value, which is 1.6 and 3.1 times higher than those of CuCuPc and CuPc, respectively, which is in line with FS and SPS results.

Quantification of built-in electric field. The IEF magnitude of CuNiPc, CuCuPc and CuPc was calculated by using the following equation (3)[28]:

$$F_S = \left(-\frac{2V_S\rho}{\varepsilon\varepsilon_0}\right)^{\frac{1}{2}} \qquad (3)$$

Where F_s represents the internal electric field magnitude, Vs is the surface potential, ρ is the surface charge density, ε is the low-frequency dielectric constant, and ε_0 is the permittivity of free space.

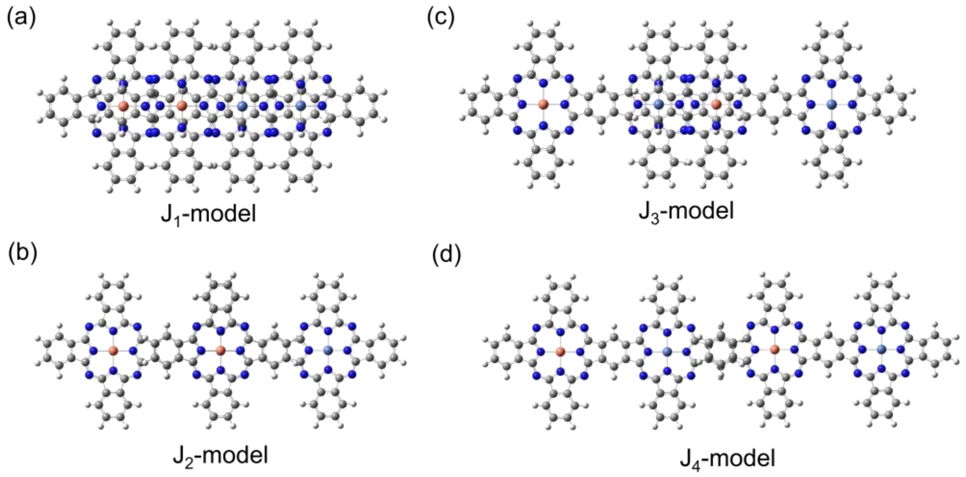

Figure S29 Different dimer configurations of the CuNiPc J-aggregates investigated in this study: J_1-model (a), J_2-model (b), J_3-model (c), and J_4-model (d).

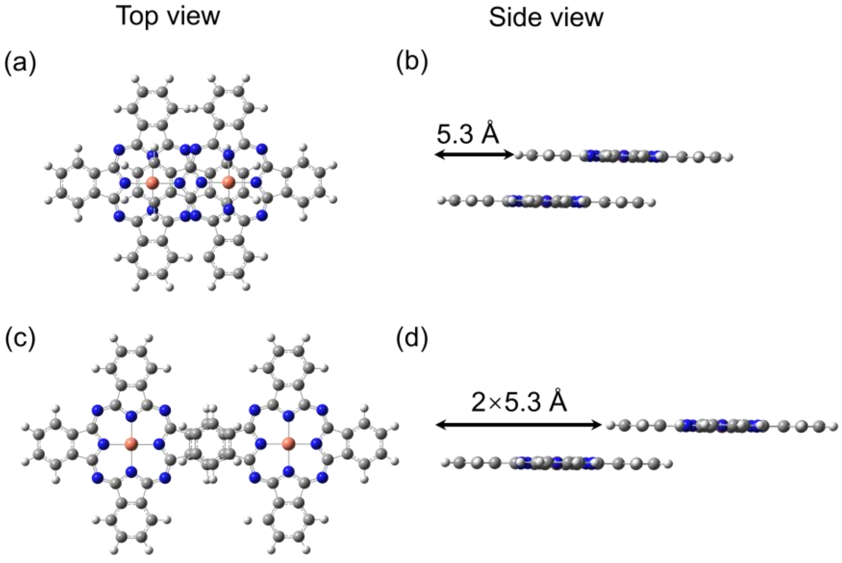

Figure S30 Different dimer configurations of the CuPc J-aggregates investigated in this study: J_1-model (a, b) and J_2-model (c, d).

Note: Two molecular stacking configurations are constructed based on the displacement difference of the stable conformations for the dimer of CuPc, namely J_1 and J_2-models. The displacement of two CuPc molecules are 5.3 Å and 10.6 Å in J_1-model and J_2-model, respectively, while there is less overlap of the electron cloud density between the molecules due to larger displacements in J_2-model. Thus, the conformation of J_2-model and CuPc(J_2)/$BiVO_4$ can be neglected in the subsequent calculations.

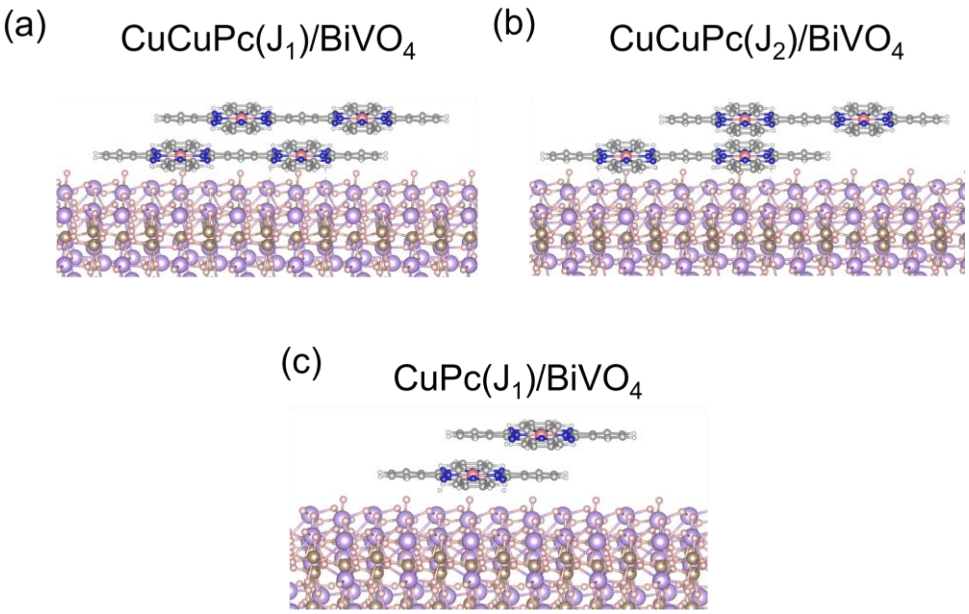

Figure S31 Side view of the DFT optimized (a) CuCuPc(J_1)/$BiVO_4$, (b) CuCuPc(J_2)/$BiVO_4$ and (c) CuPc(J_1)/$BiVO_4$ heterojunctions, respectively.

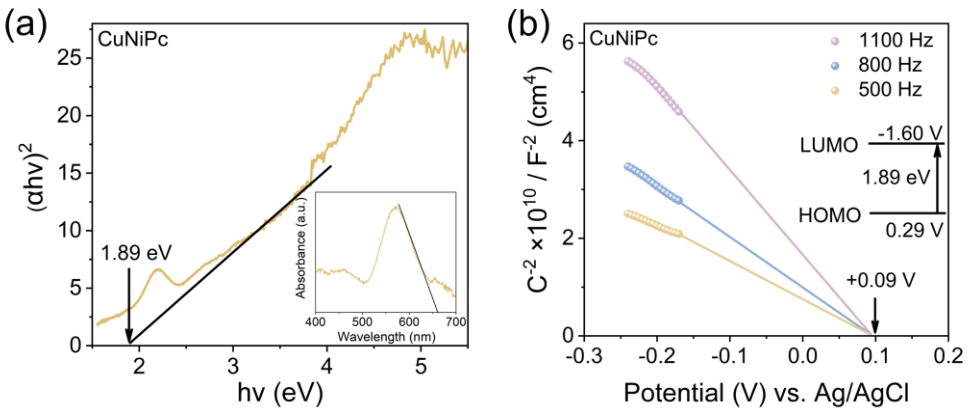

Figure S32 (a) Tauc plot of CuNiPc with the corresponding UV-vis diffuse reflectance spectrum as an inset, (b) Mott-Schottky plot of CuNiPc.

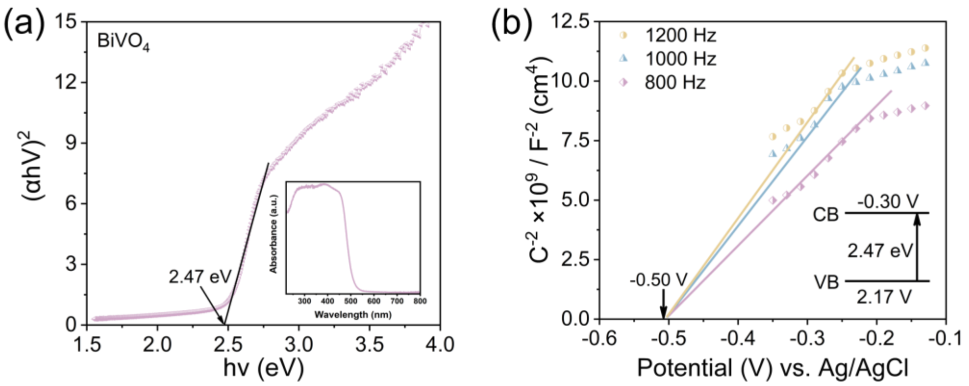

Figure S33 (a) Tauc plot of $BiVO_4$ with the corresponding UV-vis diffuse reflectance spectrum as an inset, (b) Mott-Schottky plot of $BiVO_4$.

Note: The band gap was estimated by following Tauc's relation equation (4)[29]:

$$(\alpha h\nu)^2 = \alpha_0(h\nu - E_g) \quad (4)$$

where h is Plank constant, ν is frequency of incident light, α_0 is absorption coefficient, E_g is optical band-gap.

The flat-band potential of a semiconductor can be estimated by the Mott-Schottky plot[30]:

$$E(NHE, pH = 7) = E_{Ag/AgCl} + 0.197\ V \quad (5)$$

The bandgap energies (E_g) and band structures of $BiVO_4$ and CuNiPc were measured with UV-vis DRS spectra and Mott-Schottky measurements. According to the Tauc plots, the E_g of CuNiPc and $BiVO_4$ are determined to be 1.89 and 2.47 eV. Meanwhile, the flat-band potentials for CuNiPc and $BiVO_4$ are determined to be 0.29 and -0.30 V (vs Ag/AgCl), respectively. In combination of the MS plot and Tauc plots, it can be deduced that the VB of $BiVO_4$ and LUMO level of CuNiPc locates at +2.17 and -1.60 V (vs. NHE, pH=7).

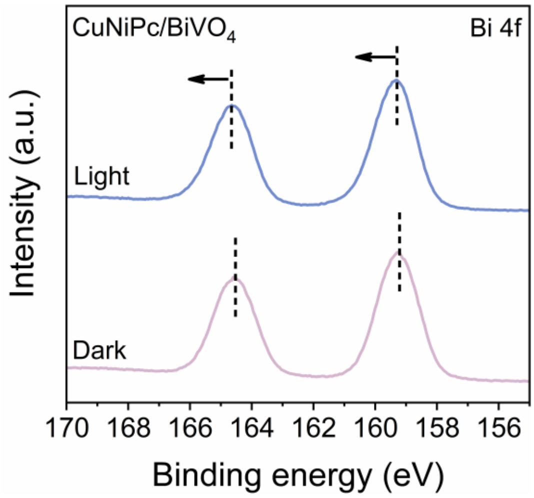

Figure S34 In-situ irradiated XPS analyses of Bi 4f over $CuNiPc/BiVO_4$ heterojunction before and after light irradiation.

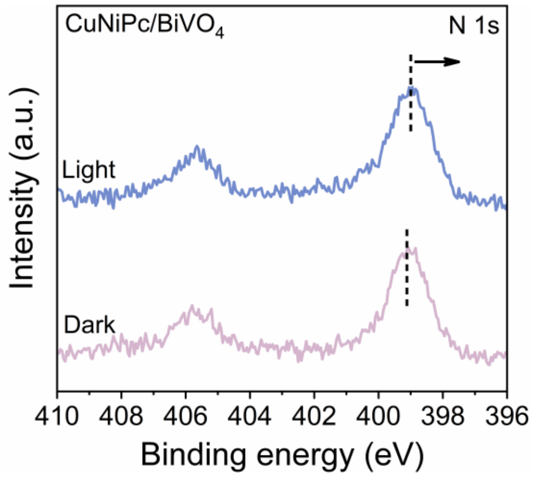

Figure S35 In-situ irradiated XPS analyses of N 1s over $CuNiPc/BiVO_4$ heterojunction before and after light irradiation.

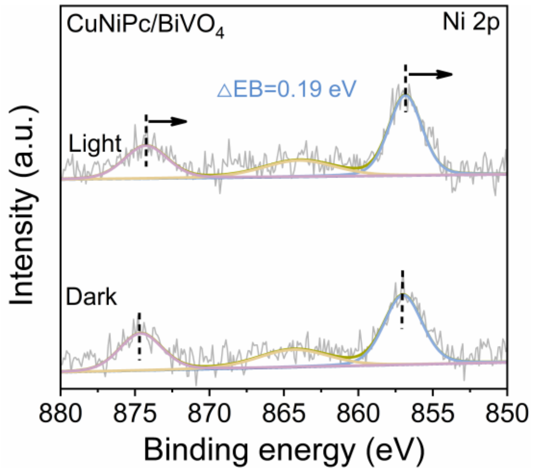

Figure S36 In-situ irradiated XPS analyses of Ni 2p over CuNiPc/$BiVO_4$ heterojunction before and after light irradiation.

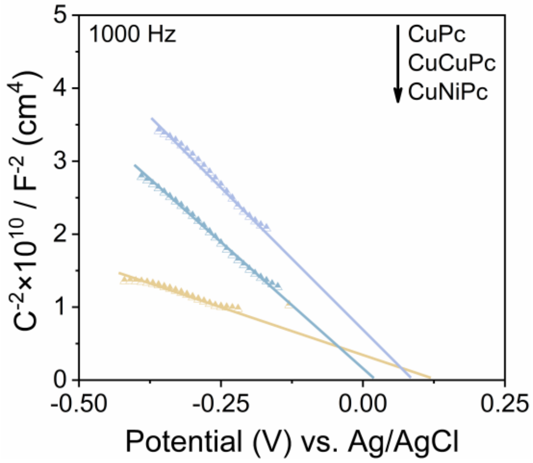

Figure S37 Mott-Schottky curves of CuNiPc, CuCuPc and CuPc collected at 1000 Hz with Ag/AgCl as the reference electrode.

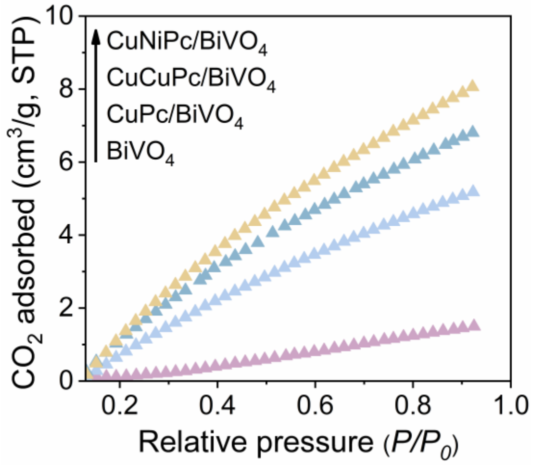

Figure S38 CO_2 adsorption isotherms of CuNiPc/$BiVO_4$, CuCuPc/$BiVO_4$, CuPc/$BiVO_4$ and $BiVO_4$.

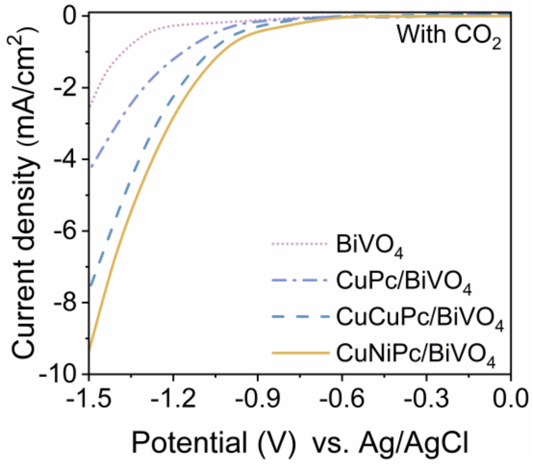

Figure39 Electrochemical reduction curves of CuNiPc/$BiVO_4$, CuCuPc/$BiVO_4$, CuPc/$BiVO_4$ and $BiVO_4$ in the CO_2-saturated electrolyte.

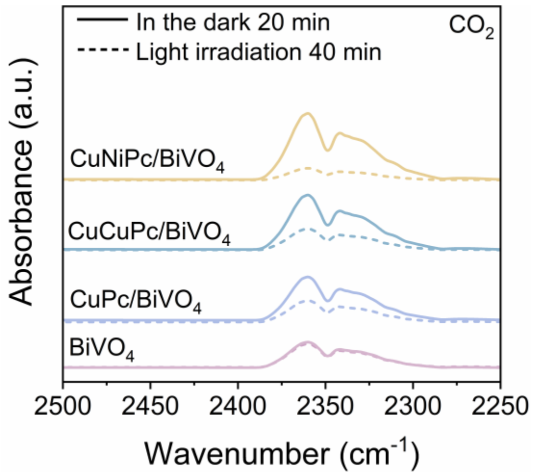

Figure S40 In-situ DRIFTS of CuNiPc/$BiVO_4$, CuCuPc/$BiVO_4$, CuPc/$BiVO_4$ and $BiVO_4$ in dark and light irradiation.

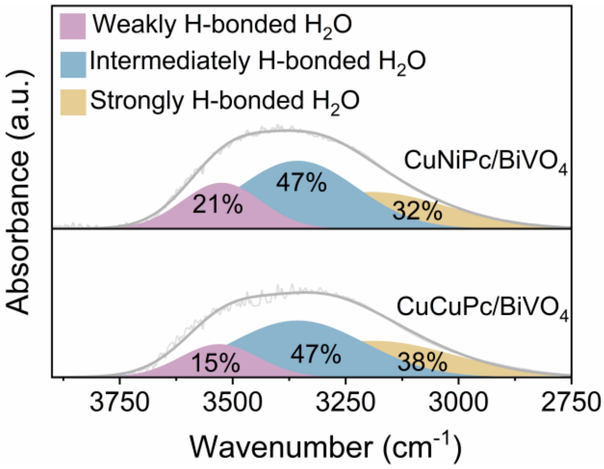

Figure S41. The corresponding O-H stretching peaks on CuNiPc/$BiVO_4$ and CuCuPc/$BiVO_4$ surfaces.

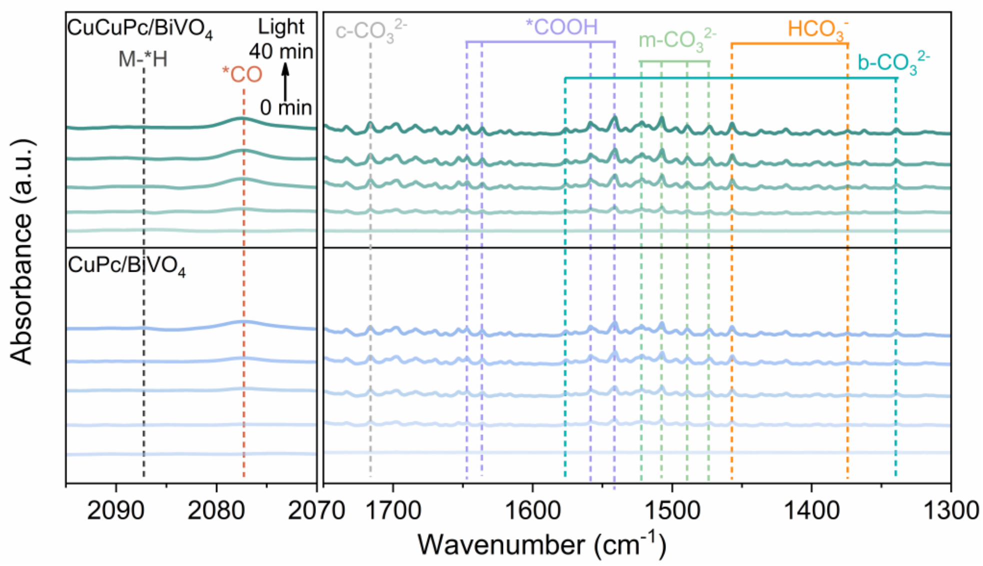

Figure S42 In-situ DRIFTS for the detected intermediates over CuCuPc/$BiVO_4$ and CuPc/$BiVO_4$ under light irradiation.

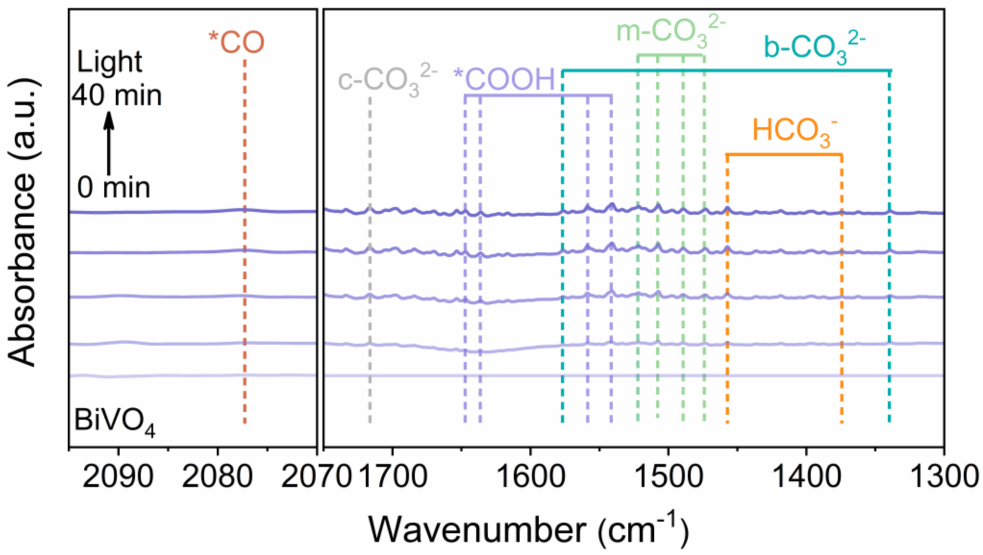

Figure S43 In-situ DRIFTS for the detected intermediates over $BiVO_4$ under light irradiation.

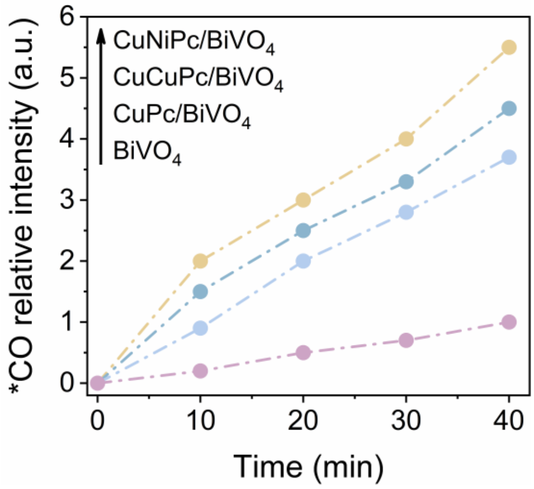

Figure S44 Relative peak intensity of CO intermediates in CuNiPc/$BiVO_4$, CuCuPc/$BiVO_4$, CuPc/$BiVO_4$ and $BiVO_4$.

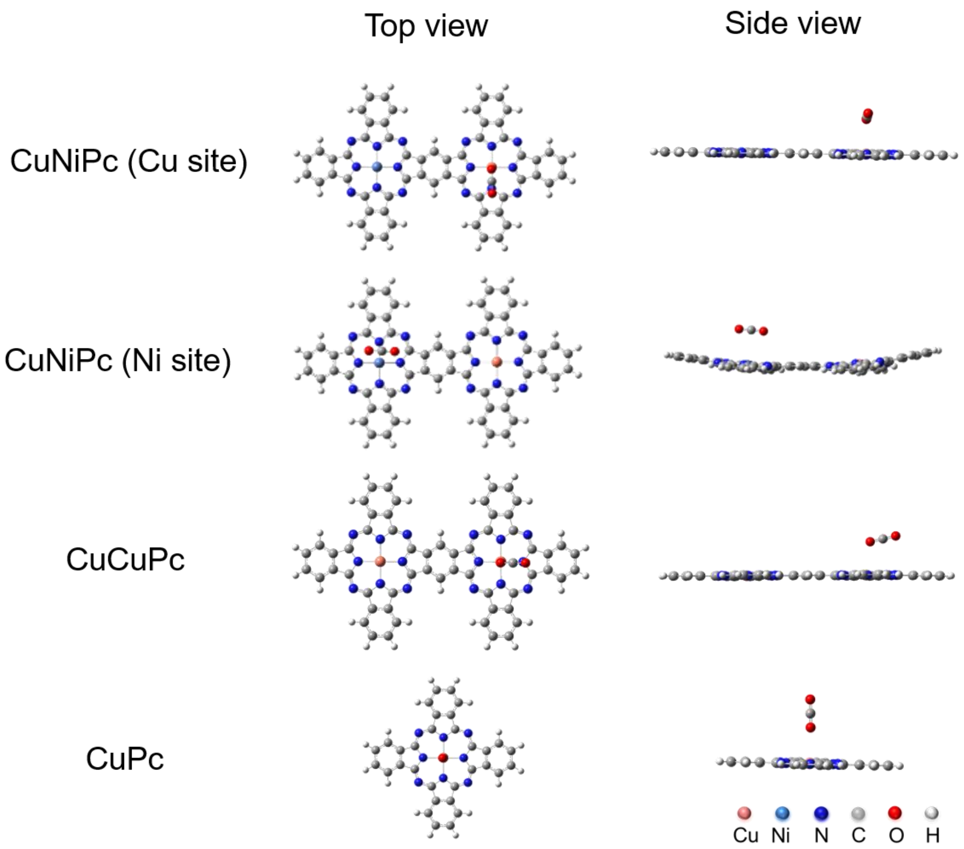

Figure S45 CO_2 absorption models on metal sites of CuNiPc, CuCuPc and CuPc by using DFT calculations.

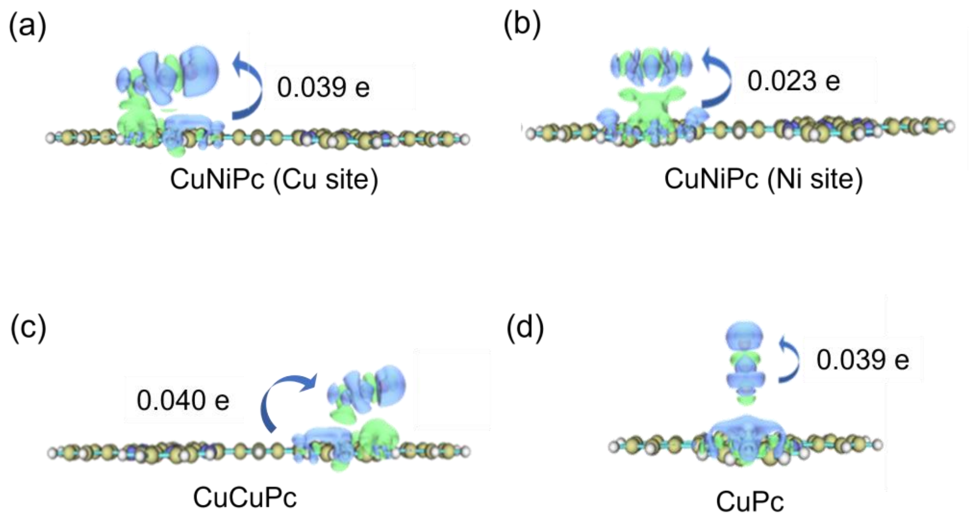

Figure S46 The calculated charge density difference images of CO_2 adsorption models on Cu site of CuNiPc (a), Ni site of CuNiPc (b), Cu sites of CuCuPc (c) and CuPc (d), respectively.

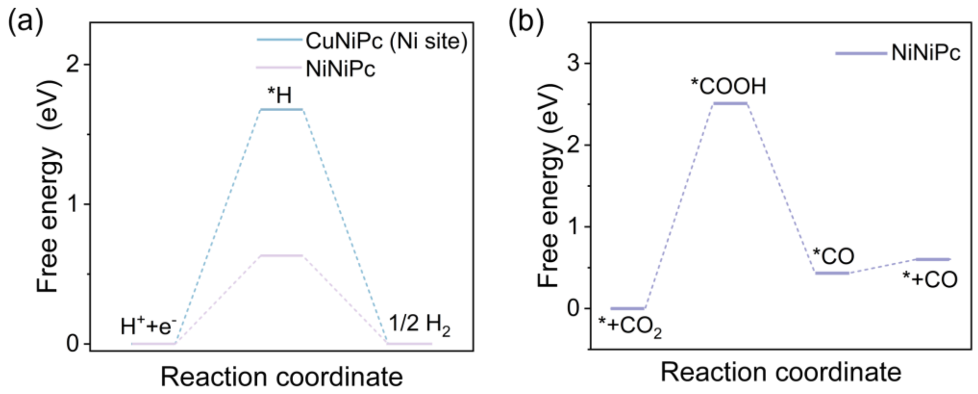

Figure S47 (a) Gibbs free energy diagram of H^+ reduction to H_2 at Ni sites over CuNiPc and NiNiPc. (b) Gibbs free energy diagram of CO_2 reduction to CO on NiNiPc.

Supporting Tables

Table S1 EXAFS fitting parameters at the Cu and Ni K-edge for various samples.

**Samples**	**Shell**	***CN***[a]	***R*(Å)**[b]	σ^2(Å^2)[c]	ΔE_0(eV)[d]	***R* factor**
Cu foil	Cu-Cu	12.0	2.54	--	--	0.001
CuPc	Cu-N	4.0	1.93	0.0017	0.1	0.017
$CuNiPc/BiVO_4$	Cu-N	3.1	1.93	0.0027	9.7	0.010
	Cu-N-Cu	3.2	2.84	0.0028	8.8	
Ni foil	Ni-Ni	12.0	2.48	--	--	0.002
NiPc	Ni-N	4.0	1.88	0.0017	0.1	0.012
$CuNiPc/BiVO_4$	Ni-N	3.3	1.89	0.0031	3.0	0.019
	Ni-N-Ni	2.8	2.89	0.0124	-6.0	

[a]*CN*, coordination number; [b]*R*, the distance to the neighboring atom; [c]σ^2, the Mean Square Relative Displacement (MSRD); [d]ΔE_0, inner potential correction; *This value was fixed during EXAFS fitting, based on the known structure of Cu and Ni. Fitting range: 3.0 ≤ *k* (/Å) ≤ 12.4 and 1.4 ≤ *R* (Å) ≤ 3.0 (Cu foil); 2.8 ≤ *k* (/Å) ≤ 10.8 and 1.0 ≤ *R* (Å) ≤ 2.9 (Cu sample); 2.4 ≤ *k* (/Å) ≤ 12.6 and 1.0 ≤ *R* (Å) ≤ 2.0 (CuPc); 3.0 ≤ *k* (/Å) ≤ 12.4 and 1.4 ≤ *R* (Å) ≤ 3.0 (Ni foil); 2.8 ≤ *k* (/Å) ≤ 10.8 and 1.0 ≤ *R* (Å) ≤ 2.9 (Ni sample); 2.4 ≤ *k* (/Å) ≤ 12.6 and 1.0 ≤ *R* (Å) ≤ 2.0 (NiPc).

Table S2 Composition analysis of the as-prepared Cu_xNi_yPc obtained from ICP-OES.

Samples	Cu (wt%)	Ni (wt%)
Cu_3Ni_1Pc	5.78	3.11
Cu_2Ni_1Pc	5.21	3.82
$Cu_{1.5}Ni_1Pc$	4.46	4.12
Cu_1Ni_1Pc	3.63	5.38

Table S3 Comparison of photocatalytic CO_2 reduction performance for CuNiPc/$BiVO_4$ with other single-atom supported catalysts under similar reaction conditions.

Photocatalyst	Light source	Experimental conditions	Production rate (μmol g^{-1} h^{-1})	CO selectivity (%)	Ref.
CuNiPc/$BiVO_4$	300 W Xe lamp	10 mg cat<br>10 mL H_2O	CO: 106.3<br>CH_4: 1.02	96.3	This work
$Co_1Ag_{(1+n)}$-PCN	300 W Xe lamp	10 mg cat<br>6 mL CH_3CN/4 mL H_2O	CO: 11.7<br>CH_4: 1.2	71	[31]
Ag@CV-KCN	300 W Xe lamp<br>AM 1.5G	10 mg cat<br>H_2O vapor	CO: 57.7<br>CH_4: 1.78	89	[32]
PtCu-crCN	300 W Xe lamp	25 mg cat<br>H_2O vapor	CO: 11.7<br>CH_4: 3.0	60	[33]
Cu SAs/TiO_2	300 W Xe lamp	10 mg cat<br>H_2O vapor	CO: 65.8	100	[34]
Mn_1Co_1/CN	300 W Xe lamp	50 mg cat<br>2 mL H_2O	CO: 47	100	[35]
0.7Ni-5OB-CN	300 W Xe lamp	50 mg cat<br>10 mL H_2O	CH_4: 8.7<br>CO: 22.1	39	[36]
$Au_{SA}/Cd_{1-x}S$	300 W Xe lamp	30 mg cat<br>H_2O vapor	CO: 32.2<br>CH_4: 11.3	42	[37]
CoRu-HCNp	300 W Xe lamp	25 mg cat<br>H_2O vapor	CO: 27.3<br>CH_4: 0.6	92	[38]
IL/Co-bCN	300 W Xe lamp	50 mg cat<br>3 mL H_2O	CO: 40.5<br>CH_4: 6.3	62	[39]
Ni-SA-x/ZrO_2	Xe lamp	10 mg cat<br>H_2O vapor	CO: 11.8	92.5	[40]

Table S4 Summary of TA spectra fitting parameters for CuNiPc, CuCuPc and CuPc at 600 nm.

Samples	A_1	τ_1 (ps)	A_2	τ_2 (ps)	A_3	τ_3 (ps)	τ_{ave} (ps)
CuNiPc	-0.33	0.54	-0.46	8.14	-0.16	150.32	130.33
CuCuPc	-0.25	0.56	-0.47	7.80	-0.20	151.32	135.29
CuPc	-0.49	0.81	-0.41	7.72	-0.07	125.11	90.94

Note: The decay curves fitting of phthalocyanines, heterojunctions, and $BiVO_4$ were tri-exponential fitting using the following equation (6, 7)[41]:

$$I(t) = A_1 e^{-t/\tau_1} + A_2 e^{-t/\tau_2} + A_3 e^{-t/\tau_3} \tag{6}$$

$$\tau_{ave} = \frac{(A_1\tau_1^2 + A_2\tau_2^2 + A_3\tau_3^2)}{A_1\tau_1 + A_2\tau_2 + A_3\tau_3} \tag{7}$$

τ_1, τ_2 and τ_3 can be assigned to the trapping process of carriers, the recombination of the excited excitons and trap-mediated recombination process, respectively.

Table S5 Summary of TA spectra fitting parameters for NiNiPc and NiPc at 610 nm.

Samples	A_1	τ_1 (ps)	A_2	τ_2 (ps)	A_3	τ_3 (ps)	τ_{ave} (ps)
NiNiPc	-0.37	0.57	-0.41	7.68	-0.15	133.10	114.97
NiPc	-0.48	0.25	-0.30	3.18	-0.10	90.56	81.26

Table S6 Summary of TA spectra fitting parameters for CuNiPc/$BiVO_4$ and CuPc/$BiVO_4$ at 600 nm.

Samples	A_1	τ_1 (ps)	A_2	τ_2 (ps)	A_3	τ_3 (ps)	τ_{ave} (ps)
CuNiPc/$BiVO_4$	-0.38	0.98	-0.35	17.39	-0.13	300.49	260.19
CuPc/$BiVO_4$	-0.50	0.96	-0.38	9.06	-0.08	168.12	131.98

Table S7 Summary of TA spectra fitting parameters for CuNiPc/$BiVO_4$, CuPc/$BiVO_4$ and $BiVO_4$ at 460 nm.

Samples	A_1	τ_1 (ps)	A_2	τ_2 (ps)	A_3	τ_3 (ps)	τ_{ave} (ps)
CuNiPc/$BiVO_4$	-0.41	0.26	-0.02	3.67	-0.03	444.85	438.95
CuPc/$BiVO_4$	-0.19	0.73	-0.13	1.85	-0.14	261.51	258.84
$BiVO_4$	-0.36	0.13	-0.18	2.00	-0.18	168.74	166.53

Table S8 Calculated charge carrier densities of CuNiPc, CuCuPc and CuPc.

Samples	Slope	ND (cm^{-3})
CuNiPc	2.50×10^{10}	1.94×10^{19}
CuCuPc	6.78×10^{10}	7.20×10^{18}
CuPc	7.94×10^{10}	6.21×10^{18}

Note: The charge carrier density was calculated based on the Mott-Schottky results using the following equation (8, 9)[42]:

$$\frac{1}{C^2} = \left(\frac{2}{N_D e \varepsilon_0 \varepsilon}\right)\left[(E - E_{FB}) - \frac{kT}{e}\right] \quad (8)$$

$$N_D = \frac{2}{e\varepsilon_0\varepsilon}\left[\frac{d\left(\frac{1}{C^2}\right)}{dE}\right]^{-1} \quad (9)$$

where C, N_D, E and E_{FB} represent the charge capacitance, carrier density, applied potential and flat band potential, respectively. The constants e, ε_0 and ε correspond to the charge constant (1.60×10^{-19} C), vacuum permittivity (8.85×10^{-12} F m^{-1}) and relative permittivity (2.9 for phthalocyanine) respectively.[43] Additionally, k and T denote the Boltzmann constant (1.38×10^{-23} J K^{-1}) and temperature, respectively.

References

1. M. Liu, N. Li, S. H. Cao, et al., A “Pre-Constrained Metal Twins” Strategy to Prepare Efficient Dual-Metal-Atom Catalysts for Cooperative Oxygen Electrocatalysis. Adv. Mater. 2022, 34 (7), 2107421. DOI: https://doi.org/10.1002/adma.202107421.
2. W. W. Zhu, S. Q. Liu, K. M. Zhao, G. Y. Ye, K Huang, and Z. He, Revealing a Double-Volcano-Like Structure-Activity Relationship for Substitution-Functionalized Metal-Phthalocyanine Catalysts toward Electrochemical CO_2 Reduction. Small. 2024, 20 (4), 2306144. DOI: https://doi.org/10.1002/smll.202306144.
3. S. Dohm, A. Hansen, M. Steinmetz, S. Grimme, M. P. Checinski, Comprehensive Thermochemical Benchmark Set of Realistic Closed-Shell Metal Organic Reactions. J. Chem. Theory Comput. 2018, 14 (5), 2596-2608. DOI: 10.1021/acs.jctc.7b01183.
4. C. Adamo, V. Barone, Toward reliable density functional methods without adjustable parameters: The PBE0 model. J. Chem. Phys. 1999, 110 (13), 6158-6170. DOI: 10.1063/1.478522.
5. M. Ernzerhof, G. E. Scuseria, Assessment of the Perdew–Burke–Ernzerhof exchange-correlation functional. J. Chem. Phys. 1999, 110 (11), 5029-5036. DOI: 10.1063/1.478401.
6. G. A. Petersson, A. Bennett, T. G. Tensfeldt, M. A. Al-Laham, W. A. Shirley, and J. Mantzaris, A complete basis set model chemistry. I. The total energies of closed-shell atoms and hydrides of the first-row elements. J. Chem. Phys. 1988, 89 (4), 2193-2218. DOI: 10.1063/1.455064.
7. W. R. Wadt, P. J. Hay, J. Ab initio effective core potentials for molecular calculations. Potentials for main group elements Na to Bi. J. Chem. Phys. 1985, 82 (1), 284-298. DOI: 10.1063/1.448800.
8. P. J. Hay, W. R. Wadt, Ab initio effective core potentials for molecular calculations. Potentials for the transition metal atoms Sc to Hg. J. Chem. Phys. 1985, 82 (1), 270-283. DOI: 10.1063/1.448799.
9. S. P. Wang, Y. Wang, F. Y. Chen, et al. Accurate Analysis of Anisotropic Carrier Mobility and Structure–property Relationships in Organic BOXD Crystalline Materials. Front. Chem. 2021, 9 775747, Original Research. DOI: 10.3389/fchem.2021.775747.
10. S. Yanagisawa, T. Yasuda, K. Inagaki, Y. Morikawa, K. Manseki, and S. Yanagida, Intermolecular Interaction as the Origin of Red Shifts in Absorption Spectra of Zinc-Phthalocyanine from First-Principles. J. Phys. Chem. A 2013, 117 (44), 11246-11253. DOI: 10.1021/jp407608w.

11. H. J. Zhu, D. Y. Zhang, E. Y. Feng, and X. W, Sheng, Effects of aggregation on the structures and excited-state absorption for zinc phthalocyanine. Phys. Chem. Chem. Phys. 2023, 25 (15), 10278-10287. DOI: 10.1039/D2CP04372F.

12. H. X. Zhong, M. Ghorbani Asl, K. H. Ly, et al., Synergistic electroreduction of carbon dioxide to carbon monoxide on bimetallic layered conjugated metal-organic frameworks. Nat. Commun. 2020, 11 (1), 1409. DOI: 10.1038/s41467-020-15141-y.

13. Y. Yue, P. Y. Cai, K. Xu, et al. Stable Bimetallic Polyphthalocyanine Covalent Organic Frameworks as Superior Electrocatalysts. J. Am. Chem. Soc. 2021, 143 (43), 18052-18060. DOI: 10.1021/jacs.1c06238.

14. L. G. C. Rego, V. S. Batista, Quantum Dynamics Simulations of Interfacial Electron Transfer in Sensitized TiO_2 Semiconductors. J. Am. Chem. Soc. 2003, 125 (26), 7989-7997. DOI: 10.1021/ja0346330.

15. S. G. Abuabara, L. G. C. Rego, V. S. Batista, Influence of Thermal Fluctuations on Interfacial Electron Transfer in Functionalized TiO_2 Semiconductors. J. Am. Chem. Soc. 2005, 127 (51), 18234-18242. DOI: 10.1021/ja055185u.

16. W. Li, L. G. C. Rego, F. Q. Bai, et al., What Makes Hydroxamate a Promising Anchoring Group in Dye-Sensitized Solar Cells? Insights from Theoretical Investigation. J. Phys. Chem. Lett 2014, 5 (22), 3992-3999. DOI: 10.1021/jz501973d.

17. L. J. He, W. Wei, J. Chen, et al., The effect of D–[De–π–A]$_n$ (n = 1, 2, 3) type dyes on the overall performance of DSSCs: a theoretical investigation. J. Mater. Chem. C. 2017, 5 (30), 7510-7520. DOI: 10.1039/C7TC02499A.

18. K. F. Zhang, J. Xu, T. R. Yan, et al., Molecular Modulation of Sequestered Copper Sites for Efficient Electroreduction of Carbon Dioxide to Methane. Adv. Funct. Mater. 2023, 33 (17), 2214062. DOI: https://doi.org/10.1002/adfm.202214062.

19. L. Jiang, M. Z. Gu, S. R. Zhao, et al., Regulating the Active Sites of Metal–Phthalocyanine at the Molecular Level for Efficient Water Electrolysis: Double Deciphering of Electron-Withdrawing Groups and Bimetallic. Small. 2023, 19 (11), 2207243. DOI: https://doi.org/10.1002/smll.202207243.

20. L. Huang, R. Lu, W. C. Zhang, et al., Precisely Regulating Asymmetric Charge Distribution by Single-Atom Central Doped Ag-Based Series Clusters for Enhanced Photoreduction of CO_2 to Alcohol Fuels. Angew. Chem., Int. Ed. 2024, 63 (46), e202412964. DOI: https://doi.org/10.1002/anie.202412964.

21. N. Han, Y. Wang, L. Ma, et al., Supported Cobalt Polyphthalocyanine for High-Performance Electrocatalytic CO_2 Reduction. Chem 2017, 3 (4), 652-664. DOI: 10.1016/j.chempr.2017.08.002.

22. K. Lakshmanan, W. H. Huang, S. A. Chala, et al., Generating Multi-Carbon Products by Electrochemical CO_2 Reduction via Catalytically Harmonious Ni/Cu Dual Active Sites. Small. 2024, 20 (17), 2307180. DOI: https://doi.org/10.1002/smll.202307180.

23. K. J. Chen, M. Q. Cao, G. H. Ni, et al., Nickel polyphthalocyanine with electronic localization at the nickel site for enhanced CO_2 reduction reaction. Appl. Catal., B 2022, 306, 121093. DOI: https://doi.org/10.1016/j.apcatb.2022.121093.

24. C. Jia, X. Tan, Q. Sun, et al., Fluorine Doping-Assisted Reconstruction of Isolated Cu Sites for CO_2 Electroreduction Toward Multicarbon Products. Adv. Mater. 2025, 37 (9), 2417443. DOI: https://doi.org/10.1002/adma.202417443.

25. K. J. Chen, M. Q. Cao, Y. Y. Lin, et al., Ligand Engineering in Nickel Phthalocyanine to Boost the Electrocatalytic Reduction of CO_2. Adv. Funct. Mater. 2022, 32 (10), 2111322. DOI: https://doi.org/10.1002/adfm.202111322.

26. Y. Zhang, X. Y. Zhang, L. Jiao, Z. Meng, and H. L. Jiang, Conductive Covalent Organic Frameworks of Polymetallophthalocyanines as a Tunable Platform for Electrocatalysis. J. Am. Chem. Soc. 2023, 145 (44), 24230-24239. DOI: 10.1021/jacs.3c08594.

27. J. M. Kroon, R. B. M. Koehorst, M. van Dijk, G. M. Sanders, and E. J. R. Sudhölter, Self-assembling properties of non-ionic tetraphenylporphyrins and discotic phthalocyanines carrying oligo (ethylene oxide) alkyl or alkoxy units. J. Mater. Chem. 1997, 7 (4), 615-624. DOI: 10.1039/A605328I.

28. X. L. Zhu, Y. H. Jia, Y. H. Liu, et al., Enhancing Built-in Electric Fields via Molecular Symmetry Modulation in Supramolecular Photocatalysts for Highly Efficient Photocatalytic Hydrogen Evolution. Angew. Chem., Int. Ed. 2024, 63 (26), e202405962. DOI: https://doi.org/10.1002/anie.202405962.

29. Y. C. Yao, C. Zhu, R. L. Liu, et al., Synergistic Tri-efficiency Enhancement Utilizing Functionalized Covalent Organic Frameworks for Photocatalytic H_2O_2 Production. Small. 2024, 20 (50), 2404885. DOI: https://doi.org/10.1002/smll.202404885.

30. J. M. Wu, K. Y. Li, B. Zhou, et al., Bottom-Up Strategy to Enhance Long-Range Order of Poly (Heptazine Imide) Nanorods for Efficient Photocatalytic CO_2 Methanation. Angew. Chem., Int. Ed. 2025, 64 (10), e202421263. DOI: https://doi.org/10.1002/anie.202421263.

31. A. X. Deng, E. Zhao, Q. Li, et al., Atomic Cobalt–Silver Dual-Metal Sites Confined on Carbon Nitride with Synergistic Ag Nanoparticles for Enhanced CO_2 Photoreduction. ACS Nano 2023, 17 (12), 11869-11881. DOI: 10.1021/acsnano.3c03176.

32. Y. Zhang, M. M. Zhai, J. Liu, et al., Anchoring Ag Atom on Carbon Vacancy Enriched Carbon Nitride to Synergistically Promote CO_2 Photoredution with Water. Adv. Funct. Mater. 2025, 35 (3), 2413232. DOI: https://doi.org/10.1002/adfm.202413232.

33. L. Cheng, P. Zhang, Q. Y. Wen, J. J. Fan, and Q. J. Xiang, Copper and platinum dual-single-atoms supported on crystalline graphitic carbon nitride for enhanced photocatalytic CO_2 reduction. Chin. J. Catal. 2022, 43 (2), 451-460. DOI: https://doi.org/10.1016/S1872-2067(21)63879-2.

34. H. B. Yin, F. Dong, D. S. Wang, and J. H. Li, Coupling Cu Single Atoms and Phase Junction for Photocatalytic CO_2 Reduction with 100% CO Selectivity. ACS Catal. 2022, 12 (22), 14096-14105. DOI: 10.1021/acscatal.2c04563.

35. H. H. Ou, S. B. Ning, P. Zhu, et al., Carbon Nitride Photocatalysts with Integrated Oxidation and Reduction Atomic Active Centers for Improved CO_2 Conversion. Angew. Chem., Int. Ed. 2022, 61 (34), e202206579. DOI: https://doi.org/10.1002/anie.202206579.

36. Y. Y. Wang, Y. Qu, B. H. Qu, et al., Construction of Six-Oxygen-Coordinated Single Ni Sites on g-C_3N_4 with Boron-Oxo Species for Photocatalytic Water-Activation-Induced CO_2 Reduction. Adv. Mater. 2021, 33 (48), 2105482. DOI: https://doi.org/10.1002/adma.202105482.

37. Y. H. Cao, L. Guo, M. Dan, et al., Modulating electron density of vacancy site by single Au atom for effective CO_2 photoreduction. Nat. Commun. 2021, 12 (1), 1675. DOI: 10.1038/s41467-021-21925-7.

38. L. Cheng, X. Y. Yue, L. X. Wang, et al., Dual-Single-Atom Tailoring with Bifunctional Integration for High-Performance CO_2 Photoreduction. Adv. Mater. 2021, 33 (49), 2105135. DOI: https://doi.org/10.1002/adma.202105135.

39. Y. Liu, J. H. Sun, H. H. Huang, et al., Improving CO_2 photoconversion with ionic liquid and Co single atoms. Nat. Commun. 2023, 14 (1), 1457. DOI: 10.1038/s41467-023-36980-5.

40. X. Y. Xiong, C. L. Mao, Z. J. Yang, et al., Photocatalytic CO_2 Reduction to CO over Ni Single Atoms Supported on Defect-Rich Zirconia. Adv. Energy Mater. 2020, 10 (46), 2002928. DOI: https://doi.org/10.1002/aenm.202002928.

41. H. Song, K. Sun, H. M. Huang, et al., Integrating photochemical and photothermal effects for selective oxidative coupling of methane into C_{2+} hydrocarbons with multiple active sites. Nat. Commun. 2025, 16 (1), 2831. DOI: 10.1038/s41467-025-58101-0.

42. S. C. Wang, P. Chen, J. H. Yun, Y. X. Hu, and L. Z. Wang, An Electrochemically Treated $BiVO_4$ Photoanode for Efficient Photoelectrochemical Water Splitting. Angew. Chem. Int. Ed. 2017, 56 (29), 8500-8504. DOI: https://doi.org/10.1002/anie.201703491.

43. N. Aimai, R. D. Gould, A. M. Saleh, Space-charge-limited conductivity in evaporated α-form metal-free phthalocyanine thin films. Vacuum 1998, 50 (1), 53-56. DOI: https://doi.org/10.1016/S0042-207X(98)00014-1.
